# Disparate genetic divergence patterns in three corals across a pan-Pacific environmental gradient highlight species-specific adaptation

**DOI:** 10.1038/s44185-023-00020-8

**Published:** 2023-07-07

**Authors:** Christian R. Voolstra, Benjamin C. C. Hume, Eric J. Armstrong, Guinther Mitushasi, Barbara Porro, Nicolas Oury, Sylvain Agostini, Emilie Boissin, Julie Poulain, Quentin Carradec, David A. Paz-García, Didier Zoccola, Hélène Magalon, Clémentine Moulin, Guillaume Bourdin, Guillaume Iwankow, Sarah Romac, Bernard Banaigs, Emmanuel Boss, Chris Bowler, Colomban de Vargas, Eric Douville, Michel Flores, Paola Furla, Pierre E. Galand, Eric Gilson, Fabien Lombard, Stéphane Pesant, Stéphanie Reynaud, Matthew B. Sullivan, Shinichi Sunagawa, Olivier P. Thomas, Romain Troublé, Rebecca Vega Thurber, Patrick Wincker, Serge Planes, Denis Allemand, Didier Forcioli

**Affiliations:** 1 Department of Biology, University of Konstanz, 78457 Konstanz, Germany; 2 PSL Research University, EPHE, CNRS, Université de Perpignan, Perpignan, France; 3 Shimoda Marine Research Center, University of Tsukuba, 5-10-1, Shimoda, Shizuoka, Japan; 4 Université Côte d’Azur, CNRS, INSERM, Institute for Research on Cancer and Aging, Nice (IRCAN), Nice, France; 5 LIA ROPSE, Laboratoire International Associé Université Côte d’Azur—Centre Scientifique de Monaco, Monaco, Principality of Monaco; 6 French National Institute for Agriculture, Food, and Environment (INRAE), Université Côte d’Azur, ISA, France; 7 UMR 250/9220 ENTROPIE UR-IRD-CNRS-Ifremer-UNC, Laboratoire d’Excellence CORAIL, Université de la Réunion, St Denis de la Réunion, France; 8 PSL Research University: EPHE-UPVD-CNRS, USR 3278 CRIOBE, Laboratoire d’Excellence CORAIL, Université de Perpignan, 52 Avenue Paul Alduy, 66860 Perpignan, France; 9 Génomique Métabolique, Genoscope, Institut François Jacob, CEA, CNRS, Univ Evry, Université Paris-Saclay, Evry, France; 10 Research Federation for the study of Global Ocean Systems Ecology and Evolution, FR2022/Tara GOSEE, 3 rue Michel-Ange, 75016 Paris, France; 11 Centro de Investigaciones Biológicas del Noroeste (CIBNOR), Av. IPN 195, Col. Playa Palo de Santa Rita Sur, La Paz, 23096 Baja California Sur México; 12 Centre Scientifique de Monaco, 8 Quai Antoine Ier, MC-98000 Monaco, Principality of Monaco; 13 Fondation Tara Océan, Base Tara, 8 rue de Prague, 75 012 Paris, France; 14 School of Marine Sciences, University of Maine, Orono, 04469 ME USA; 15 Sorbonne Université, CNRS, Station Biologique de Roscoff, AD2M, UMR 7144, ECOMAP, Roscoff, France; 16 Institut de Biologie de l’Ecole Normale Supérieure, Ecole Normale Supérieure, CNRS, INSERM, Université PSL, Paris, France; 17 Laboratoire des Sciences du Climat et de l’Environnement, LSCE/IPSL, CEA-CNRS-UVSQ, Université Paris-Saclay, Gif-sur-Yvette, France; 18 Weizmann Institute of Science, Department of Earth and Planetary Sciences, 76100 Rehovot, Israel; 19 Sorbonne Université, CNRS, Laboratoire d’Ecogéochimie des Environnements Benthiques (LECOB), Observatoire Océanologique de Banyuls, Banyuls-sur-Mer, France; 20 Department of Medical Genetics, CHU Nice, Nice, France; 21 Laboratoire d’Océanographie de Villefranche, UMR 7093, Sorbonne Université, CNRS, 06230 Villefranche sur mer, France; 22 Institut Universitaire de France, 75231 Paris, France; 23 European Molecular Biology Laboratory, European Bioinformatics Institute, Wellcome Genome Campus, Hinxton, Cambridge, CB10 1SD UK; 24 Department of Microbiology and Department of Civil, Environmental and Geodetic Engineering, The Ohio State University, Columbus, OH USA; 25 Department of Biology, Institute of Microbiology and Swiss Institute of Bioinformatics, ETH Zürich, Zurich, Switzerland; 26 School of Biological and Chemical Sciences, Ryan Institute, University of Galway, University Road, H91 TK33 Galway, Ireland; 27 Department of Microbiology, Oregon State University, Corvallis, OR USA

**Keywords:** Evolutionary genetics, Population genetics, Genetic variation, Conservation biology, Ecological genetics

## Abstract

Tropical coral reefs are among the most affected ecosystems by climate change and face increasing loss in the coming decades. Effective conservation strategies that maximize ecosystem resilience must be informed by the accurate characterization of extant genetic diversity and population structure together with an understanding of the adaptive potential of keystone species. Here we analyzed samples from the Tara Pacific Expedition (2016–2018) that completed an 18,000 km longitudinal transect of the Pacific Ocean sampling three widespread corals—*Pocillopora meandrina*, *Porites lobata*, and *Millepora* cf. *platyphylla*—across 33 sites from 11 islands. Using deep metagenomic sequencing of 269 colonies in conjunction with morphological analyses and climate variability data, we can show that despite a targeted sampling the transect encompasses multiple cryptic species. These species exhibit disparate biogeographic patterns and, most importantly, distinct evolutionary patterns in identical environmental regimes. Our findings demonstrate on a basin scale that evolutionary trajectories are species-specific and can only in part be predicted from the environment. This highlights that conservation strategies must integrate multi-species investigations to discern the distinct genomic footprints shaped by selection as well as the genetic potential for adaptive change.

## Introduction

Coral reef ecosystems harbor approximately one-third of the world’s multicellular marine biodiversity^1^ despite covering only ~0.1% of the seafloor^2^. Critically, however, these ecosystems are also some of the most sensitive to climate change^3^ with decreasing global coral coverage over recent decades^4^. In line with even moderate projections of global warming, 70–90% of coral reefs may disappear in the coming decades^5,6^, jeopardizing the biological diversity they support and the more than 500 million people who rely on the services they provide^7^. To minimize further losses, and maximize their potential for recovery, the implementation of effective conservation strategies for these ecosystems is imperative^8–10^. Moreover, developing adaptation strategies and building increased reef resilience based on the natural adaptive potential of corals has become a requirement to ensure a future for coral reefs^5,9^.

A critical component underlying the success of these conservation strategies is the preservation of the reef-building corals that form the ecological and physical foundations of reef ecosystems^8,11^. Accurately characterizing the extant genetic diversity and genetic structure of these corals^12^, as determined by their connectivity^12–14^, is an essential prerequisite for planning marine reserve networks that can replenish lost genetic diversity^8^. Characterization of life history traits such as growth form and reproductive mode among other biological attributes such as acclimation and adaptation potential are also essential, as different species may have significantly disparate responses to prevailing and historical environmental conditions^15,16^.

However, unrecognized interspecific (cryptic) diversity and high intraspecific morphological plasticity, e.g., in the genera *Acropora* and *Pocillopora*^17–19^ make characterizing genetic diversity through morphological characteristics alone problematic. The accurate characterization of diversity is improved through integration of morphological data with genome-wide sequencing strategies, e.g., RAD-Seq, RNA-Seq, whole-genome/-exome shotgun sequencing^16,20^ or, more recently, target-capture of ultraconserved elements (UCE)^21–24^. Such approaches enable fine-scale resolution of genetic lineages, estimations of relatedness and divergence timings, and identification of genomic loci under selection^25^, all of which are of high value to conservation planning efforts. To best inform conservation efforts, robust genetic characterization of corals (i.e., from deep whole-genome sequencing) across large geographic ranges are therefore required. Such campaigns are however difficult to achieve due to the logistical challenge of standardized sample collection and considerable financial requirements.

Across the Pacific Ocean it is largely unknown how coral diversity is structured, how the prevailing environment has shaped evolutionary history, and whether the consequential evolutionary trajectories are shared between corals. To begin to answer these questions, we analyzed samples from the Tara Pacific Expedition^26^ that ran from 2016 to 2018 and completed an 18,000 km longitudinal transect of the Pacific Ocean, sampling three widespread corals, two scleractinian corals—*Pocillopora meandrina* and *Porites lobata*—and one hydrozoan coral—*Millepora* cf. *platyphylla*—across 33 sites from 11 islands. These species were targeted using their colony morphologies which inevitably led to the sampling of cryptic species widespread in corals. We conducted ultra-deep metagenomic sequencing of 269 coral colonies. This sequencing effort was then combined with morphological analyses and climate data to identify the presence of putative cryptic species, determine the standing genetic diversity and population structure within each characterized lineage, and reveal disparate patterns of environmentally linked genomic loci under selection. Our work demonstrates that different species are differentially shaped by the same prevailing environment and exhibit distinct genomic footprints. Such information is critical to guide ongoing and future conservation efforts.

## Results

**Fig. 1:**
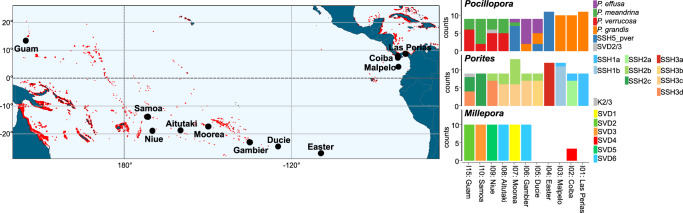
Biogeography of genetic delineations of the genera *Pocillopora*, *Porites*, and *Millepora* across their Pacific spread. The number of samples belonging to a given genetic delineation are given for each of the genera. Reference reefs from the United Nations Environment Programme—World Conservation Monitoring Center’s (UNEP-WCMP) Global Distribution of Coral Reefs dataset^111,112^ are plotted in red using reefMapMaker^113^.

Morphology-guided sampling of colonies resembling *P. meandrina*, *P. lobata*, and *Millepora* cf. *platyphylla* resulted in the collection of 106, 109, and 54 coral colonies across 11 islands, spanning 18,000 km of overwater distance (Fig. 1 and Supplementary Table 1). To resolve species identities, we designated the sampling regime as morphological-based primary species hypotheses (PSH) that we further developed into secondary species hypotheses (SSH) through an integrated taxonomic approach by analyzing genetic diversity and morphological parameters of the sampled colonies^14^. For each of the three coral genera, building on the genomic and transcriptomic sequences produced by the Tara Pacific consortium^27^, we used multiple genetic analyses to characterize the extent and distribution of extant genetic diversity.

### Genetic delineation and biogeography of *Pocillopora* spp.

**Fig. 2:**
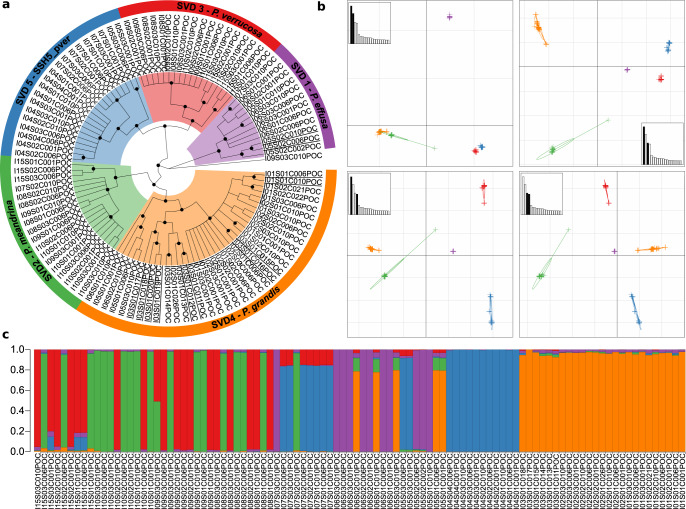
Genetic delineation of *Pocillopora* spp. **a** SVDquartet tree. Node support ≥80% from 100 bootstraps is indicated with a black dot. Samples are grouped into five clades (SVD1-SVD5) based on tree morphology and sNMF analysis which form the secondary species hypotheses (SSHs). Species names resulting from subsequent analyses (see the relevant section of this study) have been annotated. Samples not included in the sNMF analysis (due to removal of clonal/multilocus lineages) are underlined. **b** Principal component analysis (PCA) based on genome-wide SNPs. The relative eigenvalues are shown in the subplots with the displayed eigenvectors shaded black. **c** Hierarchical genetic clustering using sNMF. Bars in each column represent the contribution of predicted ancestral lineages to each sample. Samples are ordered longitudinally by island.

For *Pocillopora*, Maximum Likelihood (ML; Supplementary Fig. 1) and SVDquartet (used to estimate the species-tree topology based on SNP data under a multi-species coalescent model; Fig. 2a) trees shared a similar topology with five well-supported clades (SVD1-SVD5) containing the same constituent samples and the same single outlier sample (I09S03C010). The SVD1 clade was more distantly related to the other SVD clades and we could observe two pairs of sister clades: SVD3 & SVD5 and SVD2 & SVD4. These clades were further supported through the estimation of individual ancestry coefficients using sNMF genetic clustering and principal component analysis (PCA), which predicted five ancestral populations (K1-K5). While minimal introgression was apparent across most samples, several subsets of samples from specific islands, resolved to specific subclades of the SVD tree, displayed higher levels of introgression (e.g., K3_I15, K4_I05, and K5_I07; Fig. 2c). The one sample that grouped as an outlier in the ML and SVDquartet trees demonstrated a hybrid ancestry with a ~50/50 split of the ancestral populations otherwise associated with samples from the SVD2 and SVD3 groupings. Despite the island-specific patterns of admixture in some lineages, sNMF analysis within the identified SVD lineages identified no further genetic clustering. The SVD groupings were recapitulated in the PCA across the four highest-scoring principal components (PCs; Fig. 2b). We tested multiple SSHs by coalescence analysis using two replicate runs of BFD* with samples categorized by SVD group. The most likely species hypothesis differed between runs, but the second most likely hypothesis in both runs designated each SVD a separate species (Supplementary Table 2). We adopted this 5-species SSH as our working hypothesis. Of note, the difficulty in such assignments lies in avoiding mixing of isolated populations and species. Given that one has to drastically reduce the sampling size for this type of analysis, we chose to discard SSHs whose likelihood ranks varied between replicate runs. To assign species names to our SSH, two representatives for each SVD grouping were mapped onto the *Pocillopora* reference target-capture sequences from a previous study^28^ to then call the species-diagnostic SNPs generated therein. While SVD1-SVD4 could be assigned to the following species names: *Pocillopora* cf. *effusa* (SVD1), *Pocillopora* *meandrina* (SVD2), *Pocillopora* *verrucosa* (SVD3), and *Pocillopora* *grandis* (SVD4), SVD5 was most similar to a lineage with no formal name that is closely related to *P. verrucosa* (hereafter referred to as SSH5_pver; Supplementary Fig. 2). SVD1 was attributed the name *P*. cf*. effusa,* as *P. effusa* still lacks a formal taxonomic description. In addition, the species name attribution of each genetic lineage was further confirmed using mtORF sequences extracted from our metagenomic data and compared to previously published records^19,29–31^ (see Supplementary Table 3 and Supplementary Fig. 3). When assessing how samples from the different species were distributed across our sampling regime, we found that species exhibited region-specific distributions with each region composed of adjacent islands (Fig. 1). *P. meandrina* and *P. verrucosa* were distributed in the western Pacific, *P*. cf*. effusa* and SSH5_pver in the central Pacific, and *P. grandis* in the eastern and central Pacific. At all but the four most eastern sites (Easter, Malpelo, Coiba, and Las Perlas), multiple species were found in sympatry (Fig. 1). Notably however, *P. verrucosa* and SSH5_pver as well as *P. meandrina* and *P. grandis*, the two most genetically similar species pairings, were only found in allopatry in our samples.

### Genetic delineation and biogeography of *Porites* spp.

**Fig. 3:**
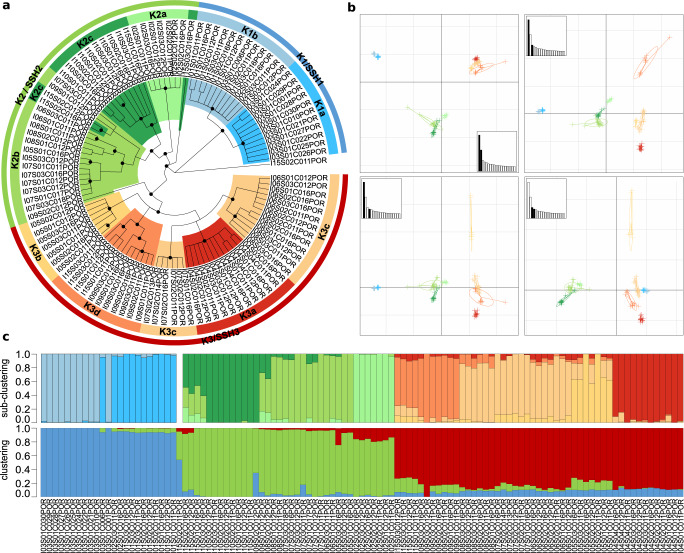
Genetic delineation of *Porites* spp. **a** SVDquartet tree. Node support ≥80% from 100 bootstraps is indicated with a black dot. Samples are grouped into three main clades (K1-K3) and two, three, and four subclades, respectively (K1a, K1b, K2a, etc.) based on tree morphology and sNMF analysis with the main clades representing the three secondary species hypotheses (SSHs; annotated). **b** Principal component analysis (PCA) based on genome-wide SNPs. The relative eigenvalues are shown in the subplots with the displayed eigenvectors shaded in black. **c** Hierarchical genetic clustering using sNMF across all samples with main clades below and subclades above. Bars in each column represent the contribution of predicted ancestral lineages to each sample. Samples are ordered by cluster and then longitudinally by island.

The ML (Supplementary Fig. 1) and SVDquartet (Fig. 3a) trees generated for *Porites* both supported the existence of three clades (SVD1-SVD3) with SVD2 and SVD3 more closely related to each other than SVD1. All samples resolved within one of these clades with the exception of sample I15S02C011 that resolved between SVD1 and the other two clades. These clades were further supported by sNMF genetic clustering and principal component analysis (PCA). The sNMF analysis suggested three ancestral populations (K1-K3) with minimal introgression apparent (Fig. 3c). Notably, there was exact agreement between SVDquartet clade placement and sNMF assignments (according to highest admixture coefficient; hereafter, SVD1 will be related to K1, SVD2 to K2, SVD3 to K3) for all but three of the samples, which exhibited significant levels of admixture (see Supplementary Table 4). Unlike in *Pocillopora*, genetic subclustering was detected within each of the sNMF groupings with two, three, and four ancestral populations predicted for K1, K2, and K3 (hereafter referred to as K1a, K1b, K2a, etc.; Fig. 3c), respectively. These subclusterings matched the subclade structure in the SVD groupings (exact match in SVD1 and SVD3, close match in SVD2; Fig. 3a) and largely demonstrated island-specific, but not endemic, patterns (Fig. 1). We tested the SSHs by coalescence analysis using the sNMF subcluster groupings (i.e., K1a, K1b, K2a, etc.). As in *Pocillopora*, the most likely species hypothesis differed between runs, but the second most likely hypothesis in both runs designated K1, K2 and K3 as distinct species. We adopted this 3-species SSH as our working hypothesis and hereafter refer to SSH1-SSH3 in reference to K1-K3. By mapping our metagenomic reads on species-diagnostic loci^32^, we were able to assign SSH1 to *Porites evermanni* and SSH2 and SSH3 to *P. lobata* (Supplementary Fig. 4). Thus, SSH2 and SSH3 putatively constitute two cryptic lineages within the *P. lobata* species. We will therefore refer to them hereafter as SSH2_plob and SSH3_plob. As for *Pocillopora*, each of the SSHs were distributed across multiple islands with one or two lineages found at each island. SSH2_plob and SSH3_plob were most widely distributed with each lineage found at seven islands (absent in only Malpelo and Las Perlas), while *P. evermanni* (SSH1) was found only at the easternmost three sites (Malpelo, Coiba, and Las Perlas; side-by-side with SSH2_plob; Fig. 1). In contrast to *Pocillopora* where genetically similar species pairings were only found in allopatry, the more genetically similar species SSH2_plob and SSH3_plob were found in widespread sympatry.

### Genetic delineation and biogeography of *Millepora* spp.

**Fig. 4:**
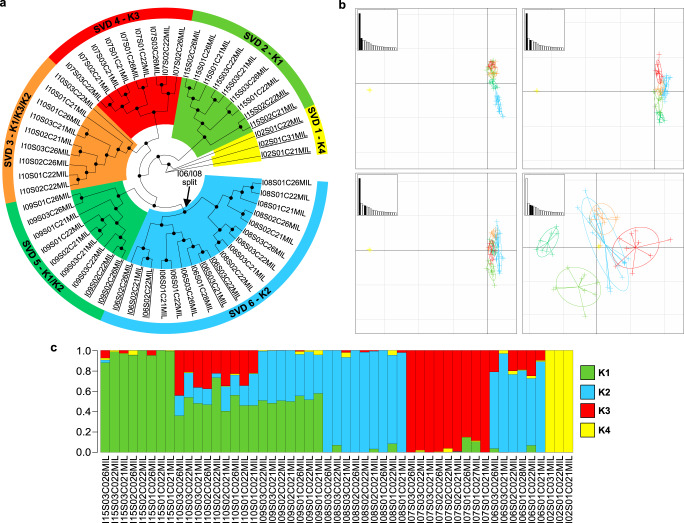
Genetic delineation of *Millepora* spp. **a** SVDquartet tree. Node support ≥80% from 100 bootstraps is indicated with a black dot. Samples are grouped into six clades (SVD1-SVD6) based on tree morphology and sNMF analysis. Samples not included in the sNMF analysis (due to the removal of clonal/multilocus lineages) are underlined. **b** Principal component analysis (PCA) based on transcriptome-wide SNPs. The relative eigenvalues are shown in the subplots with the displayed eigenvectors shaded black. **c** Hierarchical genetic clustering using sNMF. Bars in each row represent the contribution of predicted ancestral lineages to each sample. Samples are ordered longitudinally by island.

In *Millepora*, agreement in topology between the ML (Supplementary Fig. 1) and SVDquartet (Fig. 4a) trees was poor, unlike in *Pocillopora* and *Porites*. While the SVD tree showed relatively strong bootstrap values that supported the existence of six clades (SVD1-SVD6), the branches of the ML tree lacked support and distinct clades were difficult to resolve due to the low number of SNPs identified, with the exception of the *M. intricata* samples (identified as a distinct species based on field-based morphological assessment; see Methods) that were resolved separately from all other samples in both trees. The sNMF analysis predicted four ancestral populations (K1-K4) with levels of introgression varying considerably. Unlike in *Pocillopora* and *Porites*, the sNMF ancestral populations did not correspond directly to the SVD groupings, and rather, discrete combinations of the four ancestral populations resolved six groups that corresponded to the SVD clades (Fig. 4c). PC1 of the PCA analysis grouped the *M. intricata* samples separately from all others, while the remaining samples grouped according to the five remaining SVD groups across PCs 2–4 (Fig. 4b). Due to the low levels of support in the ML tree, the higher levels of introgression from predicted ancestral populations in extant samples (sNMF analysis), and the lack of direct correspondence between the sNMF ancestral populations and the SVD groupings, we considered the five non-*M. intricata* SVD groupings (SVD2-SVD6) to be representative of a single species, designated as *Millepora* cf. *platyphylla*^33^. Unlike in *Pocillopora* and *Porites*, *Millepora* SVD clades were all endemic to specific islands with the exception of SVD6. Nevertheless, a split at the basal node of the SVD6 group partitioned the samples into two clades with all members endemic to either Gambier or Aitutaki (Figs. 1 and 4a).

### Morphological analysis

In parallel to the genetic analyses, we also conducted morphological analyses that employed a multivariate suite of macromorphological measurements (see “Methods”). This served a dual purpose: first, we wanted to assess whether we could identify morphological variation between populations, and second, we wanted to assert whether such variation corresponded to the genetically identified SSHs, to provide genetic and morphological evidence of the species designations. Morphological analysis revealed structured variation in *Pocillopora* (Supplementary Fig. 5), *Porites* (Supplementary Fig. 6), and *Millepora* (Supplementary Fig. 7). We also found significant PERMANOVA results when testing the multivariate morphological characteristics against the genetic delineations, which demonstrated that some of the variation could be explained by the SSH designations (*Pocillopora*: *F*_1,75_ = 1.6669, *P* = 0.013, *n* = 77, *Porites*: *F*_2,78_ = 2.3329, *n* = 81, *P* = 0.001, and *Millepora*: *F*_4,45_ = 1.5601, *P* = 0.003, *n* = 50). Critically however, for each of the coral genera, there was no combination of morphological features that could identify the genetic resolutions; rather, each of the morphotype categorizations contained multiple SSHs. Within-genus pairwise PERMANOVA testing identified differences between all pairings except for *P. meandrina* vs. *P. verrucosa* and *P. meandrina* vs. SSH5_pver in *Pocillopora*, and SSH6 vs. SSH5, SSH6 vs. SSH2, SSH4 vs. SSH3, SSH4 vs. SSH2, SSH5 vs. SSH3, and SSH3 vs. SSH2 in *Millepora*. All within-*Porites* pairings returned significant results.

### Evolutionary history of among- and within-species genetic differentiation

**Fig. 5:**
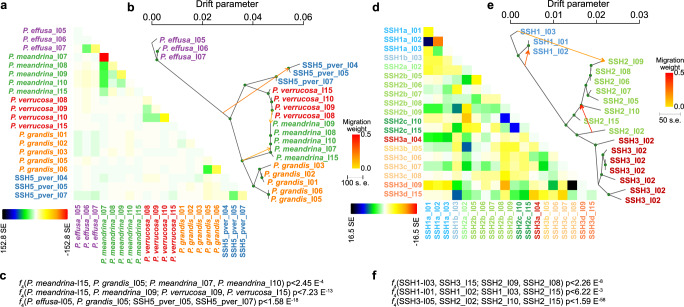
TreeMix consensus trees with migration events and residuals for *Pocillopora* and *Porites*. **a**, **d** residuals indicating potential remaining admixture between samples in *Pocillopora* and *Porites*, respectively, after accounting for the three migration events annotated in the TreeMix tree. **b**, **e** Treemix consensus trees with migration events and their weights annotated. **c**, **f** Corresponding *f*_4_ indices.

To further resolve evolutionary history in the *Pocillopora* and *Porites* species, we investigated introgression and pairwise divergence for the SSHs (Fig. 5). For *Millepora*, an insufficient number of SNPs and representative samples meant that these analyses were not conducted. In *Pocillopora*, three admixture events were predicted and verified based on significant results in the relevant *f*_4_ indices^34^ (Fig. 5b, c) where two of the admixture events were from ancestral nodes to child clades and the third was inter-SSH from *P. verrucosa*_I06 to *P. meandrina*_I09. The resulting TreeMix consensus tree showed a similar topology to that of the SVD tree with those groups from *P. effusa* more differentiated from the other SVD groups and with two pairs of sister clades formed by *P. meandrina* & *P. grandis* and *P. verrucosa* & SSH5_pver (Fig. 5b). After accounting for the three introgression events, residuals (obtained from fitting the tree model to the observed data) indicated potential remaining admixture within each of the five species (Fig. 5a). Similar to the Treemix tree, the *f*_*2*_ values (Supplementary Fig. 8A) and the distributions of genomic Weir’s *F*_ST_ values computed from 500 bp sliding windows (Supplementary Fig. 8B) recapitulated the high divergence of *P. effusa* from the other more genetically similar two species pairs (*P. meandrina* & *P. grandis* and *P. verrucosa* & SSH5_pver). Taken together, these findings reinforce the pattern in *Pocillopora* that the genetically closely related sister clade species pairings (*P. meandrina* & *P. grandis* and *P. verrucosa* & SSH5_pver) are found in our samples in allopatry rather than sympatry. In *Porites*, three admixture events were predicted and confirmed (f_4_ significant results; Fig. 5d, e) with one within-SSH, one inter-SSH, and one from an ancestral to a child clade. As for *Pocillopora*, the resulting TreeMix consensus tree topology agreed with that of the SVD tree, with *P. evermanni* (SSH1) being more differentiated compared to SSH2_plob and SSH3_plob. After accounting for the three admixture events, residuals indicated potential remaining admixture within each of the three SSHs and also between SSH2_plob and SSH3_plob (Fig. 5d). The f_2_ values (Supplementary Fig. 8C) and the distributions of Weir’s *F*_ST_ from 500 bp sliding windows (Supplementary Fig. 8D) both supported the relative divergence of *P. evermanni* (SSH1) from the other two SSHs, and in contrast to *Pocillopora*, it also showed a correlation between sympatry and genetic relatedness.

### Environment as a predictor of genetic lineage distribution

The gradientForest algorithm^35^ was used to assess whether parameters related to past sea surface temperature (SST) and depth^36^ are predictors for the geographic distribution of the genetic lineages determined for *Pocillopora* and *Porites* (Supplementary Fig. 8). For both genera, the geographic distribution of all lineages could be partially predicted based on past SST and depth with varying degrees of accuracy (Pocillopora: *P*. cf *effusa/*SVD1: *R*^2^ = 0.161, *P. meandrina*/SVD2: *R*^2^ = 0.412, *P. verrucosa*/SVD3: *R*^2^ = 0.348, *P. grandis*/SVD4: *R*^2^ = 0.565; Porites: *P. evermanni/*SSH1: *R*^2^ = 0.687, SSH2_plob: *R*^2^ = 0.290, SSH3_plob: *R*^2^ = 0.513). Among the three most contributing predictors for the geographic distribution of *Pocillopora* lineages, two were related to cold spells, namely the number of days and the mean number of days per year between 2002 and the sampling date where the SST dropped 1 °C or more below the weekly (long-term) climatology (TSA_cold_freq_sum_day: *R*^2^ = 0.095 and TSA_cold_freq_mean_day: *R*^2^ = 0.0889). The third predictor was related to SST anomalies, i.e. the maximum number of days within 1 year between 2002 and the sampling date where the SST was 1 °C or more higher than weekly climatologies (SST_anomaly_freq_max_day: *R*^2^ = 0.090). The geographic distribution of *Porites* was better predicted by SST anomalies, namely the mean number of days per year between 2002 and the sampling date where SST was 1 °C or more higher than weekly climatologies (SST_anomaly_freq_mean_day: *R*^2^ = 0.112), the mean SST anomaly (in °C) per year between 2002 and sampling date (SST_anomaly_mean_DegC: *R*^2^ = 0.095), and the seasonal mean SST (seasonal_mean_DegC: *R*^2^ = 0.095). Depth of the sampling sites was only a minor predictor for the geographic distribution of species within both genera (*Pocillopora*: *R*^2^ = 0.034, predictor 57/68, and *Porites*: *R*^2^ = 0.023, predictor 46/68).

### Correlation of genetic relatedness with geographic distance and climate

To assess for isolation-by-distance and a possible effect of temperature regime on the evolutionary trajectories of species, we separately tested for correlation between genetic distance (encoded as pairwise *F*_ST_/(1-*F*_ST_) distances generated from pairwise group *F*_ST_ distances; see “Methods”) and both, geographical distance (calculated as the overwater distance between islands) and climate (represented by past temperature difference based on island-wise Euclidean past temperature distances generated from a reduced dimensionality representation; see “Methods”)^36^. In *Pocillopora*, separate correlation analyses of genetic distance against geographical distance and temperature difference returned nonsignificant results across and within species (Supplementary Table 5). By contrast, significant results were returned for *Porites* across all species (geography, *r* = 0.397, *P* = 0.005; temperature, *r* = 0.361, *P* = 0.020) and within SSH2 (geography, *r* = 0.585, *P* = 0.036; temperature, *r* = 0.641, *P* = 0.010) and SSH3 (geography only, *r* = 0.591, *P* = 0.008). In *Millepora*, a significant result was observed for all lineages against temperature (*r* = 0.930, *P* = 0.019) but not geography (*r* = 0.722, *P* = 0.053). For each coral genus, geographical distance significantly correlated with past temperature distance (Supplementary Table 5).

### Influence of temperature regime on evolutionary trajectories

**Fig. 6:**
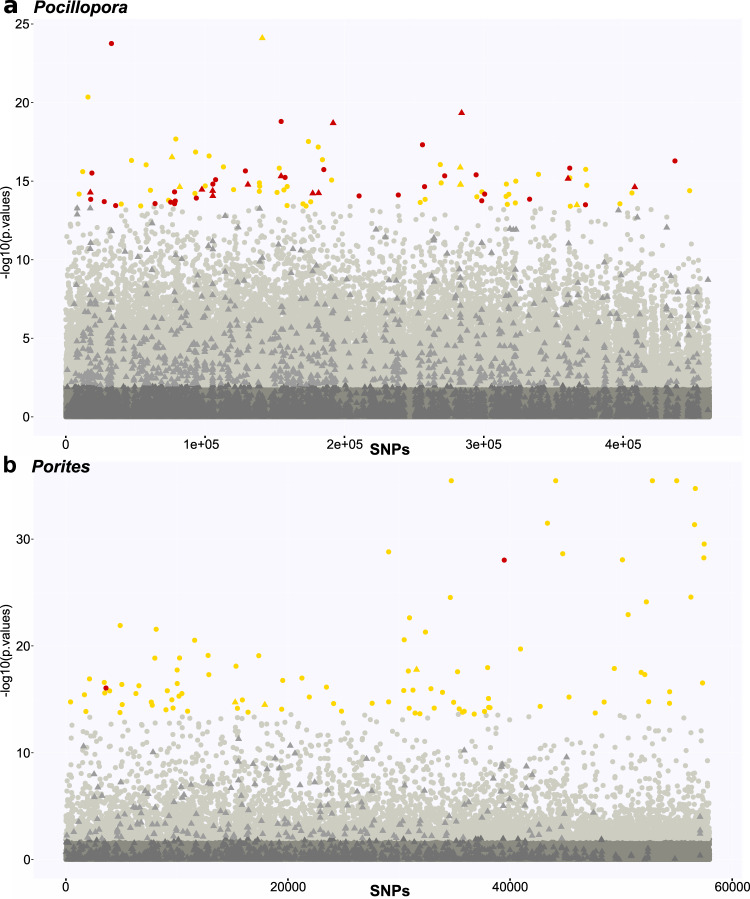
Manhattan plot displaying the association to past temperature variation of the linkage disequilibrium-filtered genome-wide SNPs computed by RDA analysis. **a** *Pocillopora*, **b** *Porites*. The *x* axis gives cumulative position in the genome with contigs sorted by decreasing size order. *P* values used to generate the *y* axis are from the RDA analysis. Circles: SNPs not included in genomic islands of differentiation between SSHs (GID); triangles: SNPs included in GIDs; dark gray: SNPs not significantly associated to past temperature variation; light gray: SNPs significantly associated to temperature variation; gold: top 100 temperature outlier SNPs; red: SNPs among the top 100 temperature outliers under divergent selection among species according to Flink analysis.

To investigate whether temperature (as a prevalent stressor of the coral metaorganism) was a potential driver of species divergence patterns in *Pocillopora* and *Porites*, we assessed whether SNPs that aligned with past temperature regimes exist. Indeed, we could identify SNPs in *Pocillopora* and *Porites* whose local allelic frequencies could be predicted by past temperature regime (i.e., temperature outlier SNPs; *Pocillopora*— 38,229 out of 461,989 SNPs (8.27%); *Porites*—6441 out of 57,966 SNPs (11.11%); at RDA *q* value <0.1 from the unlinked dataset, see “Methods”; Fig. 6). To determine those SNPs potentially involved in species divergence patterns, we queried which of the SNPs originated from a set of genomic islands of differentiation (GID) defined as the 1% of the 500 bp bins with the highest mean *F*_ST_ value among SSHs for *Pocillopora* and *Porites*. In *Pocillopora*, 20 of the top 100 temperature outlier SNPs (ranked by *P* value) were located in these GIDs (Fig. 6). By contrast, *Porites* had only 3 such SNPs in GIDs, suggesting that species differentiation is more closely correlated to thermal history in *Pocillopora* than in *Porites*. We also investigated whether this difference could be due to drift or actively maintained due to divergent adaptation. In *Pocillopora*, 21% of the whole genome was predicted to be under divergent selection (at the level of between-SSH comparisons), a considerably higher proportion than the 7% predicted in *Porites*. However, independent of this baseline difference between the two genera, in *Pocillopora* 41% of SNPs in the top 100 temperature outliers were predicted to be under selection, and this proportion grew to 61% (12 out of 20) when considering those outlier SNPs under selection and residing in GIDs, approximately double and triple that of the proportion of SNPs under divergent selection across the whole genome (21%). By contrast, in *Porites* a decrease from the 7% of SNPs under divergent selection across the whole genome was observed when considering either those outlier SNPs predicted to be under selection (2%) or those under selection and residing in GIDs (0%; 0 out of 3). This suggests that while the differentiation and distribution of the *Pocillopora* species are due to and/or maintained by divergent selection driven by temperature regimes, in *Porites* divergence among SSHs is likely due to either drift or other environmental factors (of note, many environmental factors correlate with temperature).

## Discussion

Our primary species hypothesis of one species per coral genus, based on *in situ* morphological assessment (i.e., during underwater collection), was genetically validated only for *Millepora* cf. *platyphylla*. The corals collected and putatively designated as *Pocillopora meandrina* and *Porites lobata* produced SSHs of 5 and 3 species, respectively, that were supported by multiple genetic analyses (Figs. 2–5 and Supplementary Fig. 8). Subsequent morphological analyses based on underwater colony pictures of the corals resolved a number of morphotypes (Supplementary Figs. 5–7) that showed correlation with the genetically resolved lineages in all three genera. However, multiple genetic lineages were associated with all designated morphotypes and the identification of lineage-diagnostic morphological features was not possible based on underwater colony pictures. Our finding underlines the difficulty of defining morphological criteria that can be readily derived from photographs and therefore supports the well-established existence of high morphological plasticity in corals, as exemplified by the genus *Pocillopora*^37–39^. Further, it reinforces that plasticity is a confounding factor to achieve effective taxonomic resolutions^37,40,41^ and identification of species in the field. Finally, it highlights the difficulties in conducting large geographic surveys on targeted coral species due to the putative presence of cryptic species that, although potentially common, are typically not addressed in coral reef studies^42^. Nevertheless, it also demonstrates what information can be acquired with respect to genetic divergence patterns even in the case of non-ideal sampling datasets (our dataset may comprise a ‘typical’ coral collection dataset in this regard). In the context of conservation strategies, the disparity between morphology- and genetics-based species resolution highlights the necessity for robust molecular characterization to be able to resolve species diversity.

The three genera demonstrated very different degrees of genetic diversity and connectivity despite being sampled across the same environmental range and sites. This has implications for informing conservation efforts. Our genetic analyses suggest that *Pocillopora* is characterized by a higher level of within-species connectivity than *Porites*. A correlation between genetic dissimilarity and geographic distance as well as within-species genetic structuring (inferred by sNMF genetic clustering; Figs. 1 and 3) was only detected in *Porites* in line with less resolutive past studies^43,44^, and residuals (after TreeMix consensus tree generation) gave a considerably stronger within-species migration signal in *Pocillopora* than in *Porites* (Fig. 5). Maintenance of connectivity to ensure exchange of alleles is a key aim of resilience-based conservation management strategy^8^ and has been demonstrated to facilitate robustness and recovery to stress in reef ecosystems^45–47^. Therefore, while the higher number of species resolved in *Pocillopora* may suggest a greater conservation effort is required (greater species diversity to protect), its higher connectivity, in concert with its relatively high levels of species sympatry (in certain islands all species are present when considering only two sites, e.g. I05 Ducie or I08 Aitukaki, see also^31^), mean that core levels of diversity protection and resilience maintenance may be accomplished with a relatively focused effort. However, whether observed species are truly adapted to a given location or whether their presence is the result of/dependent on migration from a seed site (see below) must be carefully considered. In contrast to *Pocillopora*, the lower levels of connectivity and relatively higher levels of genetic structure in *Porites* suggest a greater spatial area of protection would be required to conserve genetic diversity and promote resilience^16^, especially considering the geographic isolation of *P. evermanni* (SSH1) in the eastern Pacific (I01-I03; Las Perlas, Coiba, and Malpelo) and the putative presence of yet another different genetic stock within this species in the Central Pacific^32,43,44^. While *Pocillopora* and *Porites* resolved into multiple distinct species, *Millepora* was resolved as a single species with highly endemic populations containing varying levels of admixture. In the context of maintaining genetic diversity, the *Millepora* population of I10 (Samoa) would be prioritized for protection over others, as this population has the highest proportion of admixture encompassing all three of the predicted ancestral populations. A recent study^16^ also detected distinct differences in connectivity between two reef-building species of the family Pocilloporidae that may, at least partially, be due to different reproductive modes (broadcast spawner, high connectivity, low genetic structure; brooder, low connectivity, high genetic structure). The reproductive mode could explain the differences in connectivity observed here but has not been characterized for the corals sampled. Further, generalizations based on other members of the respective genera are difficult, as *Pocillopora* and *Porites* both contain species that broadcast spawn and internally fertilize (brood), with some species exhibiting location-dependent reproductive modes^48–53^. Independent of identifying a causal factor for differences in connectivity, it is clear that species- and even population-specific characterizations of connectivity are a necessity in designing effective strategies (and conservation areas/zones) for genetic diversity maintenance.

Our dataset comprising samples of multiple species of *Pocillopora* and *Porites* across the same basin-wide range has enabled us to demonstrate contrasting evolutionary histories in these two genera, despite exposure to the same past thermal, and arguably, environmental regimes. The comparison of the genetic structures obtained in the two genera from samples coming from the same sites, along with the high sequencing depth (which led to a large number of genome-wide SNPs) on which the analyses were conducted, allows to at least partially offset the low local sample sizes for some SSHs. We found that although species geographic distribution for both *Pocillopora* and *Porites* are linked to past temperature variations, the underlying mechanisms driving this distribution vastly differ. Genetic distance was correlated to past thermal distance for *Porites* but not for *Pocillopora* (Supplementary Table 5). Given the significant correlation to thermal distance in *Porites*, one might have expected past thermal variation to play a role in *Porites* speciation/species divergence. However, the relative lack of divergent selection among *Porites* species suggests that these temperature variations did not have a role in species divergence and/or species isolation in this genus, in the sense that the elicited adaptive responses (resulting in at least some of the temperature outlier SNPs revealed by the genotype/environment association analyses) did not contribute to species differentiation. Rather, it would appear that the significant correlation to temperature distance is the product of correlation between the geographic and temperature distances (*r* = 0.564, *P* = 0.000, Supplementary Table 5) with the significant genetic and geographic correlation driven by the relatively low dispersal of *Porites*^43,44,54^. In *Pocillopora*, the lack of correlation between genetic distance and thermal distance is likely a consequence of the lack of correlation between genetic and geographic distances (Supplementary Table 5), diagnostic of high levels of dispersion and subsequent migration. Migration would enable a continuous influx of individuals with minimal genetic differentiation from seed sites to destination sites. These individuals would be adapted to the seed sites and may not survive thermal events such as cold spells linked to La Niña events at the sampled sites^55^. This combined high dispersal and sensitivity to environmental stress would concurrently explain the observed high overall species sympatry, lack of genetic distance correlations, yet the considerable effect of past temperature variation on the maintenance of species divergence (if not on the speciation process itself) in *Pocillopora*^56^.

The larger effect of past temperature variation on the evolutionary histories of the *Pocillopora* species could be symptomatic of a specialization/niche adaptation strategy (as opposed to being a more environment generalist like *Porites*)^57^ whereby any perturbation from the niche environment would necessitate an adaptive response due to a limited acclimation potential^58^. This explanation would seem less parsimonious given the acknowledged high morphological plasticity of the genus considered to be a driving factor in its evolutionary success^37^. However, a detailed comparison of temperature sensitivity among the genetically defined *Pocillopora* species is still lacking. It could also be argued that the adaptive potential of *Porites* is limited, but this is unlikely given the globally recognized resilience of this genus^58–60^. The genomic data produced here can provide the starting point for future functional analyses investigating the genes and pathways associated with the temperature outlier and GID SNPs identified, thus enabling cellular insights into adaptation potential and its underlying mechanisms.

Contemporary resilience-based conservation strategies do not fully consider how corals may adapt to future challenges^5^. Given the differences between species found across the same distribution here, it is interesting to speculate on species being more or less successful under climate change. *Pocillopora* species distribution appears more linked to the environment than *Porites.* Therefore, the *Pocillopora* genus likely displays a narrower set of physiological tolerances^55,56,61^. However, *Pocillopora* is known to be a speciose genus^19^, and while some of the extant species may be lost with the continuing and growing climatic perturbations predicted in the coming decades, surviving genotypes will have been selected for the future environments^62^. Coupled with the high dispersion capability of the genus—granting access to the widest possible range of habitats—this may result in a relatively successful strategy for the *Pocillopora* genus, enabling recolonization of future reefs by its more stress-tolerant species. Conservation efforts applied to such speciose, niche-adapted, and high-dispersal coral genera may catalyze the recovery of reefs by identifying those genotypes most likely to survive future perturbations, and where possible, protecting them from further non-climatic perturbations, while facilitating their already high-dispersal capacities to ensure the seeding of as wide a range of reef ecosystems as possible^11^. In contrast, generalist corals such as the *Porites* species identified in this study may be successful in the long term as long as the magnitude of perturbations are within the buffering capacity of their wider physiological tolerances^57^. However, as these buffering capacities are surpassed, the lower dispersal capacity of these species will mean a reduced ability to recolonize alternative environments and such genera may suffer losses^5^. In contrast to the targeted strategy of the niche-adapted corals, conservation of generalist corals should likely focus on mitigating local aggravating stressors (e.g., managing pollutant and nutrient input, limiting physical disruption due to coastal development, or preventing overfishing) to maximize the buffering capacity of the corals across as many of the available genotypes as possible.

## Methods

### Sample collection and environmental metadata acquisition

In the present study, we used corals which were sampled at 11 islands (Islas de las Perlas, Coiba, Malpelo, Rapa Nui, Ducie, Gambier, Moorea, Aitutaki, Niue, Upolu, and Guam) encompassing 32 sites (~3 sites per island) across an 18,000 km longitudinal transect from July 2016 to January 2017 during the first year of the *Tara* Pacific Expedition^26^. Coordinates and associated depth of the sampled sites and the number of samples collected are detailed in Supplementary Table 1. The mean depth at which the samples were collected was 9.3 ± 3.9 m (interquartile: 6.2–12 m). Three target species were sampled based on coral colony morphology: *Pocillopora meandrina*, *Porites lobata*, and *Millepora* cf. *platyphylla*. In addition, at Coiba (I02S01), three colonies of *Millepora intricata*^33^, that displayed a distinct branching morphology, were collected. Where possible, at each site, a minimum of three coral colonies were sampled and photographed as detailed in ref. ^36^ for a total of 106, 109, and 57 colonies of *P. meandrina*, *P. lobata*, and *Millepora* spp., respectively (Supplementary Table 1). Coral fragments were preserved in 15 ml of Lysing Matrix A beads (MP Biomedicals, Santa Ana, CA, USA) with 10 ml of DNA/RNA Shield buffer (Zymo Research, Irvine, CA, USA) and stored at −20 °C onboard the *Tara* vessel. The sampling, handling, nucleic acid extractions, and sequencing of coral colonies used in this study, along with the collection of their associated contextual metadata, are shortly described here, but their full descriptions can be found in two Tara Pacific data publications^27,36^.

Past sea surface temperature (SST) variations (such as, among other descriptors, the frequency, length, and intensity of hot and cold anomalies, etc.) supposedly had an impact on colony survival and recruitment, and therefore, structure the current genetic diversity in these species^63^. Therefore, at each sampling site, ocean skin temperature (11 and 12 μm spectral bands longwave algorithm) was extracted from 1 km resolution level-2 MODIS-Aqua and MODIS-Terra from 2002 to the sampling date and from level-2 VIIRS-SNPP from 2012 to the sampling date. Day and night overpasses were used to maximize data recovery. Following recommendations from NASA Ocean Color (OB.DAAC), only SST products of quality 0 and 1 were used. The nine closest pixels to the sampling sites were extracted. All the extracted pixels from the 3 platforms were then averaged daily to obtain daily SST averages and standard deviation time series for each sampling site, from 2002 to the sampling date. Different measures of the variation of these SSTs have been computed from these averages (as detailed in ref. ^36^) and used in the present study for the genotype/environment association and Gradient Forest analyses (see below).

### DNA/RNA isolation, library preparation, and sequencing

For the *Pocillopora*, *Porites,* and *Millepora* colonies, coral fragments were disrupted using a high-speed homogenizer FastPrep-24 5 G Instrument (MP Biomedicals, Santa Ana, CA, USA). For DNA isolation, a preliminary enzymatic digestion of 1 h at 37 °C was applied on 500 µl of coral tissue with 50 µl of lysozyme (50 µl 10 mg/ml), 3 µl of mutanolysine (50 KU/ml), and 3 µl of lysostaphine (4 KU/ml). DNA and RNA were isolated with the Quick-DNA/RNA Kit (Zymo Research, Irvine, CA, USA). DNA and RNA were quantified on a Qubit 2.0 fluorometer with Qubit dsDNA BR (Broad range) and HS (High sensitivity) Assays and Qubit RNA HS Assay (ThermoFisher Scientific, Waltham, MA, USA), respectively.

For DNA, library preparation protocols were constructed to generate narrow-sized libraries around 300–800 bp using the NEBNext DNA Modules Products or the NEBNext Ultra II DNA Library prep kit (New England Biolabs, MA, USA). For *Millepora*, RNA libraries were prepared following the TruSeq Stranded mRNA sample preparation protocol. Extracted RNA (1 µg) was subjected to polyA+ selection using oligo(dT) beads, then converted into single-stranded cDNA using random hexamer priming. The second strand was generated to create double-stranded cDNA, purified by 1.8× AMPure XP bead cleanups, and ligated to TruSeq RNA barcoded adapters (Illumina, San Diego, CA, USA) or NEXTflex DNA barcoded adapters (Bioo Scientific, Austin, TX, USA). DNA and RNA libraries were sequenced using 151 bp paired-end read chemistry on a NovaSeq or HiSeq4000 Illumina sequencer (Illumina, San Diego, CA, USA) with a targeted yield of 10^8^ reads per sample.

For both DNA and RNA, we removed short (<30 bp length) reads, low-quality nucleotides (Q score <20), adapter/primer sequences, and read pairs that mapped to the Enterobacteria phage PhiX174 genome (GenBank: NC_001422.1). For RNA, read pairs that mapped to ribosomal sequences were removed using SortMeRNA v2.1^64^.

The raw sequences used for this study are deposited at the European Nucleotide Archive (ENA) at the EMBL European Bioinformatics Institute (EMBL-EBI) under the Tara Pacific Umbrella BioProject PRJEB47249. Additional methodologies for DNA isolation and sequencing are available in ref. ^27^.

### SNP calling and filtering

For *Pocillopora* and *Porites*, we identified a set of genome-wide single-nucleotide polymorphisms (SNPs) by mapping the metagenomic reads to the *Pocillopora* cf*. effusa* and *Porites* *lobata* genomic references generated as part of the Tara Pacific expedition^65^ using the Genome Analysis Toolkit software (GATK, v3.7.0)^66^. We followed a modified version of the best practices protocol for variant discovery with GATK with manual filtering of the resulting variants. The following protocol was carried out for each species independently.

We aligned Illumina-generated 150-bp paired-end metagenomic reads sequenced from each colony to the predicted coding sequences of the *Pocillopora* cf*. effusa* and *Porites* *lobata* coral host reference genomes using Burrows–Wheeler Transform Aligner (BWA-mem, v0.7.15) with the default settings^67^. A read was considered a host contig if its sequence aligned with ≥95% sequence identity and with ≥50% of the sequence aligned. Host-mapped reads were sorted and filtered to remove sequences which contained >75% of low-complexity bases and <30% high-complexity bases using SAMtools v1.10.2^68^ with the resultant bam files visualized using the Integrated Genomics Viewer^69^. The reference genomes were indexed with picardtools v2.6.0 (https://broadinstitute.github.io/picard/) using *CreateSequenceDictionary* before performing local realignment around small insertions and deletions (indels) using GATK’s *RealignerTargetCreator* and *IndelRealigner* to reduce false-positive variant identification and represent indels more parsimoniously.

After sample pre-processing, we called DNA variants (SNPs and indels) individually for each colony (GATK, *HaplotypeCaller*), generating one Genomic Variant Call Format (GVCF) file per sample. We then combined all per-colony GVCF files for a given island (ca. 9 files per island) into a single, multi-sample GVCF file (GATK, *CombineGVCFs*). This resulted in the generation of 11, island-specific combined GVCF files for each species. We then consolidated these 11 multi-sample GVCFs into a GenomicsDB database which allowed for subsequent variant calling across all island cohorts through a joint genotyping analysis (GATK, *GenotypeGVCFs*). Because the *GenotypeGVCFs* tool is capable of handling any ploidy level (or a mix of ploidies) intelligently, we did not specify the ploidy level in our function call. This analysis resulted in the generation of a single, “raw” Variant Call Format (VCF) file for each species in which all colonies were jointly genotyped. Because well-curated filtering resources necessary for GATK’s *Variant Quality Score Recalibration* (VQSR) tool were not available for either coral species, we filtered the raw VCF files manually using VCFtools v0.1.12 ^70^ keeping only biallelic sites with minor allele frequencies ≥0.05, site-quality scores ≥30, and no missing data across colonies. This produced the “linked” datasets (5,937,714 and 1,971,638 variant sites with minimum read depth coverages ≥16 and 17 for *Pocillopora* and *Porites*, respectively) from which the SNPs were used for the identification of genomic islands of differentiation and the genomic signatures of selection. In order to avoid variant site linkage effects in downstream genetic analyses, we additionally filtered variants for linkage disequilibrium. We used the “prune” add-on feature in BCFtools v1.11^71,72^ to discard variants with a linkage disequilibrium (*r*^2^) ≥ 0.2 in a window of 1000 sites. This resulted in the “unlinked” datasets (347,243 and 183,222 variant sites for *Pocillopora* and *Porites*, respectively). The SNPs from this curated set were used to identify Secondary Species Hypotheses (SSHs) using individual-based phylogenies, genetic hierarchical clustering and species trees, and to test for population structure, admixture, and Genotype Environment associations, as described below.

For the detection of clonal lineages, we had to further reduce the size of these unlinked datasets to avoid unreasonable computing times. This was performed in *Pocillopora* by first further filtering on linkage disequilibrium (*r*^2^ ≥ 0.02) by BCFtools to a 27,382 variant site dataset for a first round of clone detection, and then by partitioning the individuals by SVDquartet lineages (see below). The coalescent testing of SSHs needed a further size reduction of the datasets, again on linkage disequilibrium, and keeping only one individual per putative genetic lineage per island (two replicates of 25 individuals and 636 variant sites and of 24 individuals and 1032 variant sites for *Pocillopora* and *Porites*, respectively).

Due to the absence of a reference genome at the time of SNP calling for *Millepora* cf. *platyphylla*, a ‘target gene’ approach was followed. Trimmed RNA-Seq metatranscriptomic sequences were subjected to de novo whole transcriptome assembly using Trinity^73^, after being further reduced to 23,755,336 reads by in silico normalization. The Trinity assembly produced 302,299 transcripts, which were then clustered into 40,560 unigenes (i.e., uniquely assembled transcripts) with an N50 of 1053 bp. Based on BUSCO, the assembled transcriptome was largely highly complete with 85.6% of the ortholog genes from the Eukaryota database being present with low fragmentation (3.6%), missing (10.8%), and duplication (16.7%) metrics. Within this transcriptome, we identified *Millepora* orthologs for an arbitrary set of stress and/or environment response genes from scleractinians and actinarians or genomic fragments from *Pocillopora* and *Porites* that contain temperature outlier SNPs obtained within the present study. The *Millepora* orthologs were obtained using QuickParanoid that takes a collection of files produced by InParanoid^74^ as input and finds ortholog clusters among species. The procedure used strictly followed the instructions published at http://pl.postech.ac.kr/QuickParanoid/. Briefly, for a given set of target genes, QuickParanoid first preprocesses each InParanoid output file by using BLASTall and then computes ortholog clusters. As for the other coral genera, metagenomic reads were aligned to this set of target genes using Burrows–Wheeler Transform Aligner (BWA-mem, v0.7.15) with the default settings^67^. Host-mapped reads were then sorted and processed using SAMtools v1.10.2^68^ to generate respective bam files, and SNPs were identified as detailed above using GATK. The list of orthologs for the *Millepora* transcriptome contigs that contained SNPs is part of the resource files and scripts deposited in ref. ^75^. The SNP dataset was then filtered for biallelic status, SNPs only, quality, missingness, and minimum allele frequency with the same thresholds as for the other coral genera resulting in a final set of 446 variant sites. One individual with an excess of missing data (I09S03C026) was not included in the sNMF analysis. Filtering on linkage disequilibrium (*r*^2^ > 0.2) for further genetic analyses reduced this dataset to 243 unlinked variant sites in 56 individuals.

### Species delimitation overview

Here we followed the integrative taxonomy approach^14^ by treating our morphology-guided sampling of the three coral species as primary species hypotheses (PSHs) from which we developed secondary species hypotheses (SSHs) through analysis of genetic diversity and colony macromorphology. We then formally tested the SSHs using coalescent analysis^25^.

### Genetic delineation of species

To obtain an initial impression of the genetic diversity contained in the *Pocillopora* and *Porites* samples, we built an individual-based Maximum Likelihood phylogenetic tree using RAxML v8.2.12^76^. For this, the “unlinked SNPs” VCFs were translated to relaxed Phylip format using PGDSpider^77^, and raxmlHPC-PTHREADS was run on 40 cores, with a GTRCAT model and the number of bootstrap replicates determined by the autoFC option. The best ML tree with support value was then transferred in Newick format to MEGA X^78^ for graphical annotation.

A species tree was then built using SVDquartets^79^ as implemented in PAUP*^80^ v4.0a152 for all three genera. This non-Bayesian approach infers the relationship among quartets of taxa under a coalescent model and uses this information to build the species tree. Parallel computing on 50 threads was performed to analyze all possible quartets and 100 bootstrap replicates. The bootstrap consensus tree was then transferred to MegaX for graphical annotation.

To select which of the SVDquartet clades formed pertinent SSH, a hierarchical genetic clustering was also performed using the program sNMF^81^ with the snmf function from the LEA v2.8.0^82^ R package. However, as this analysis is sensitive to clonality (B. Porro, pers. comm.), we first identified clonal lineages in the *Pocillopora*, *Porites,* and *Millepora* “unlinked” datasets using the R package Rclone v1.0.2^83^ to identify the pairwise threshold distance between clonal individuals, and then used the mlg.filter function from the R package poppr v2.8.6^84,85^ to assign individuals to clonal, or, more properly, multilocus lineages (MLL). We ran this clonal analysis on all samples for each of the 3 genera. In *Pocillopora* and *Porites*, due to a considerable lineage-correlating variation in pairwise distances, we also ran further analyses on SVD-grouping-defined subsets of samples. This analysis highlighted the presence of six MLL shared among 14 specimens in *Pocillopora* and five MLL shared among 12 specimens in *Millepora*. No shared MLL were found among *Porites* (Supplementary Table 3). The subsequent sNMF analyses were performed keeping only one ramet per clonal genet except for the 3 *M. intricata* samples that were identified as being a single MLL. sNMF determines the optimal number of ancestral populations from which the actual dataset could be issued through admixture. These analyses were performed on 50 cores and 10 repetitions for a number of possible ancestral populations that varied from 1 to 20, with an alpha parameter value of 10. The best number of ancestral clusters was determined by the entropy criterion, and a bar chart representing the individual admixture coefficient from each of these ancestral clusters was produced. To test for possible subclustering, a second round of sNMF analyses was performed separately on each of the ancestral clusters identified in the first round (with the same analysis parameters). As a further visualization of the genetic diversity within each of the 3 genera, we performed principal component analyses (PCA) based on individual genome-wide genotypes using the glPca function of the adegenet v1.7-15R package^86,87^. For *Pocillopra* and *Porites*, sNMF clustering and SVDquartet species-tree topology then guided the construction of secondary species hypotheses to be tested by coalescent analysis using Bayes factor delimitation with genomic data (BFD*)^88,89^. BFD analyses were not conducted in *Millepora* due to the low number of SNPs. The BFD* analyses were performed in BEAST2 2.6.2^90^ following BFD* 2017 tutorial recommendations (https://beast2.blogs.auckland.ac.nz/tutorials/), with 48 path sampler sets of 500,000 MCMC repetitions, to sample in 10,000,000 MCMC iterations of SNAPP^89^ for *Pocillopora* and 750,000 MCMC repetitions and 1,000,000 MCMC iterations of SNAPP for *Porites*. These numbers of iterations were determined as minimal for convergence by first running standard SNAPP analyses in Beast2. Following the BFD* tutorial recommendations, the priors for the runs were computed from the tree height, for the estimation of Yule model birth rate, and the mean within-species divergence, as theta estimator, from the RAxML trees, with values of respectively 0.151 and 0.115 for *Pocillopora* and 0.156 and 0.107 for *Porites*, respectively. To select for the most pertinent SSH, the BFD* likelihood ranking of the SSH was performed twice in each genus on a duplicate set of individuals for the same variant sites.

### Morphology analysis

To investigate the degree to which morphology may support or confound the genetics-based SSHs, we categorized the samples into morphotypes through morphological analysis and contrasted these groupings with the genetic resolutions. The *M. intricata* samples were excluded from the analysis. Photographs of entire colonies were taken for all samples using an underwater camera with 20 × 20 cm quadrats as a scale (photographs available online at https://store.pangaea.de/Projects/TARA-PACIFIC/Images/). Collection of morphological parameters was conducted using ImageJ v1.50e^91^, the image annotation plugin objectj v1.05j (https://sils.fnwi.uva.nl/bcb/objectj/), and custom macros with the photos corrected for perspective using the “interactive perspective” plugin with the quadrat as a reference.

The following morphological parameters were collected. For *Pocillopora*: branch tips density (calculated from the number of tips divided by the projected surface of the whole colony), tip length polyline (a line connecting the two extremities of the top of a branch following the curvature of the tip; *n* = 3), tip length ratio (the ratio between a straight line connecting the extremities of the top of a branch and the tip length polyline to detect the degree of meander shape of the branch tip; *n* = 3), tip width (*n* = 3), and colony roundness (the difference between the maximum and minimum diameters, and branch spacing between a randomly selected center branch and six adjacent branches; *n* = 6). For *Porites*: colony diameter, projected surface, growth form (columnar, massive or encrusting), lobe density (computed by the number lobes divided by the area of a 20 × 20 cm quadrat drawn on the colony photo using the ImageJ rectangle tool), and presence of ridges (present or absent). For *Millepora*: growth form (encrusting, laminar, columnar, branching columnar), column or plate density (computed by the number of columns or plates within the 20 × 20 cm quadrat), length of columns or plates (the length of a straight line connecting the extremities of the top of a column/plate; *n* = 1–6, when present), and width of columns/plates (*n* = 1–6, when present).

Morphometric analysis of the morphological parameters was conducted in R V4.1.0 using the same approach for all three genera. Unsupervised Random Forest analysis (randomForest::randomforest) was used to generate a proximity matrix upon which hierarchical clustering was performed to identify morphotypes (stat::hclust, factoextra::fviz_dend) with a threshold set so that the number of clusters equated to the number of genetically derived SSHs (*Pocillopora* and *Porites*), or the six SVD groupings (*Millepora*). The Gini coefficient was tested to measure the contribution of morphological variables in the homogeneity of the nodes of the decision trees. The proximity matrix was used as input to generate a principal coordinate analysis ordination (PCoA; stats::cmdscale) to visualize variance in morphology across the samples in accordance with the genetic lineage assignments. In addition, PERMANOVA (pairwiseAdonis::pairwise.adonis2; 999 permutations; *P*-adjusted Bonferroni correction applied; https://github.com/pmartinezarbizu/pairwiseAdonis) was used to test whether variance in morphology could be significantly explained by the genetic delineations.

### Identification of *Pocillopora* and *Porites* SSH taxonomies

To assign species names to our SSH, two representatives for each SVD were mapped on the 2068 *Pocillopora* target-capture reference sequences from ref. ^28^ using BWA^67^. Samples were then genotyped for the 1559 unlinked diagnostic SNPs generated therein. Genotypes with a minimum read depth (DP) of 12× and nonsignificant strand biases (SP < 13) were called and filtered with BCFtools v1.11^71,72^. Between 1489 and 1524 SNPs were retrieved per sample. The resulting VCF was then combined with the one used for species delimitation in ref. ^28^, and assignment tests were performed from *K* = 2 to *K* = 30 using the program sNMF^81^ with the snmf function from the LEA^82^ R package, as previously described. From that, SSH correspondence with genomic species hypotheses (GSH) from ref. ^28^, and thus current taxonomy, was retrieved. Taxonomy was further confirmed by analysis of mitochondrial open reading frame (mtORF) sequences extracted from the metagenomic reads of the sampled *Pocillopora* colonies. Host metagenomic reads were mapped against a curated collection of species-diagnostic mtORF profiles^29,37^ using the Burrows–Wheeler Transform Aligner (BWA-mem, v0.7.15) with default settings^67^. Mapped reads were then sorted and processed using SAMtools (v1.10.2) to generate respective bam files^71^. Mapped reads were further filtered to retain only sequences that aligned to a given mtORF reference with ≥95% of sequence identity and with ≥80% of the sequence aligned. For each colony, a consensus mtORF sequence was generated from the aligned reads using the SAMtools *mpileup* feature followed by multiple sequence alignment in MUSCLE (v3.8.1551). Finally, colony consensus sequences and reference mtORF sequences^31^ were aligned with MAFFT version 7.49^92^, and a maximum likelihood phylogeny was performed in MegaX version 10.1.5^93^ with bootstrap replicates to produce an mtORF phylogeny. A similar approach was applied to *Porites*, by mapping the metagenomic reads of one individual per genetic subcluster within each SSH to the *P. lobata* ortholog sequences of three species-diagnostic single-copy nuclear loci from ref. ^32^ (MM100, MM32, and ATPaseB). The orthologs of these three loci were identified in the Tara Pacific *Porites* *lobata* reference genome^65^ by BLAST from the sequences of an Hawaiian *P. evermanni* from^32^. SNPs were called with SAMtools *mpileup* and allelic variants sequences were obtained for each of these loci for each sample using the BCFtools consensus function on the resulting VCF file. The resulting fasta files were then added to the sequences from ref. ^32^ and processed following the protocols detailed therein to produce a POFAD genotype network^94^. Without a doubt, thorough species naming is a critical part of evolutionary and conservation biology^95,96^. However, it is equally important to acknowledge the limitations of morphology-targeted sampling approaches: our sampling did not include all known taxonomic diversity within the *Pocillopora* and *Porites* genera, and we also missed a good number of species types locations. For this reason, we kept our taxonomic resolution to the level offered by genetic-based analyses of described species and abstained from hypothetical and possibly misguided cryptic lineage namings. Insufficient SNPs and absence of the relevant resources limited such an analysis for *Millepora*.

### Among- and within-species genetic differentiation

To further resolve evolutionary history in the *Pocillopora* and *Porites* species designations, we investigated introgression and pairwise divergence for the SSHs. Following ref. ^97^, introgression among lineages was computed with TreeMix 1.13^98^. The unlinked SNP dataset VCF was converted to TreeMix format using vcftools^70^ and PLINK^99^. The individuals were regrouped in populations according to their SSH and islands of origin, and the optimal number of admixture events was estimated at this level. For this, we ran TreeMix 10 times for 0 to 10 events and estimated the optimal number of events using the optM R package^100^. We then ran TreeMix 100 times for this optimal number of admixture/introgression events to produce a consensus tree and bootstrap values using the BITE 1.2.0008 R package^101^. The residual covariance matrix was estimated for the optimal number of admixture/introgression events and the consensus tree using TreeMix. The occurrence of the admixture/introgression events were verified by computing the relevant *f*_4_ indices^34^ and correlation in genetic content was further quantified by calculating pairwise *f*_2_ distances using AdmixTools2 (admixtools 2.0 R package, https://uqrmaie1.github.io/admixtools/index.html) after converting the unlinked SNP dataset VCF to the BED format using vcftools^70^ and PLINK 1.90b6.21^99^.

To quantify and characterize genomic divergence between the SSHs within *Pocillopora* and *Porites,* we computed pairwise Weir and Cockerham^102^ *F*_ST_ along the genome (500 bp sliding window) with vcftools 0.1.17^70^.

### Environment as a predictor of genetic lineage distribution

The algorithm gradientForest was used to assess whether temperature-related parameters and sampling depth could be predictors for the geographic distribution of the genetic lineage distribution found in *Pocillopora* and *Porites*. The analysis was conducted in R (v4.2.2) using the library gradientForest (v0.1-32)^35^. Abundance data of the lineage at each sites were normalized using the logarithm of the abundance plus the minimum abundance observed (log(y + c) where c is the minimum positive abundance y), according to ref. ^38^. Sea surface temperature-related variables, described in ref. ^36^ and the mean depth at each sampling site, calculated from individual depth data available in ref. ^36^ were used as predictors. The gradient forest model was computed using 300 trees, a maximum of level of split of 2 (calculated following^38^), a correlation threshold of 0.5, and 101 bins as recommended in ref. ^38^).

### Correlation of genetic lineages with geographic distance and past thermal regime

To assess for isolation-by-distance and a possible effect of temperature regime on the evolutionary trajectories of the species, we conducted correlation analyses sensu^103^. For *Pocillopora* and *Porites*, samples were again grouped according to their SSH and islands of origin (i.e. SVD4_I01, SVD4_I02… etc.) while *Millepora* samples were grouped according to island of origin. Within each genus, pairwise group *F*_ST_ distances were generated using vcftools 0.1.17^70^ from the “unlinked” SNPs dataset and used to generate pairwise matrices of *F*_ST_/(1-*F*_ST_). These distances, representative of genetic dissimilarity, were then tested for correlation against distance matrices representing geographic and past temperature (environmental) distances using the mantel function from the vegan 2.5-6 R package^104^. Geographic distances were based on GPS coordinates of islands (estimated by an average of the site coordinates). Euclidean past temperature distances were generated from a multiparameter past temperature dataset collected for each of the 38 sampled sites as part of the *Tara* Pacific consortium (as detailed in ref. ^36^) and used in a site-wise manner after conducting PCA to reduce its dimensionality from 63 initial parameters to the 5 highest-scoring PCs that cumulatively accounted for >80% of the observed variance (using R package FactoMineR v2.4)^105^.

### Effect of environment on SSH divergence

To assess the influence of the environment (using past temperature variations as a proxy) on divergence between the SSHs in *Pocillopora* and *Porites*, we first used the reduced dimension past temperature variation dataset (see above) to identify temperature outlier SNPs whose allelic frequencies were linked to past temperature variation using redundancy analysis (RDA) following the exact protocol of Capblancq and Forester^106^. For this, the unlinked SNP dataset had first to be converted to ped format using vcftools. From this RDA, we identified temperature outlier SNPs, i.e., those SNPs whose allelic frequency distributions among populations were best explained by the local past temperature variation, as the 100 SNPs with the associated lowest *P* values (respectively *P* < 2.15 × 10^−4^ in *Pocillopora* and *P* < 1.72 × 10^−3^ in *Porites*). We then defined a set of genomic islands of differentiation (GIDs) as the 1% of the 500 bp bins with the highest mean *F*_ST_ value after conducting a further pairwise sliding window *F*_*ST*_ analysis as detailed above but with samples grouped according to SSH for each of the genera. We compared the proportion of these temperature outlier SNPs that were found in GIDs (see above) between the two genera. Finally, we investigated the proportion of the temperature outlier SNPs, and in particular those located in GIDs, that were predicted to be under divergent selection using the program FLink v1.0^107^. FLink operates on a dataset of linked loci and is based on the F-model which measures differences in allele frequencies (*F*_ST_) that are partitioned into locus- and population-specific components reflecting selection and drift, respectively. FLink structures samples in a hierarchical model with the level of “population” nested in “group” and “group” nested in “higher hierarchy”. We equated “group” to the SSH and “population” to island of collection for the samples, and generated allele counts from the “linked SNPs” dataset as input using ATLAS^108^. To reduce computational complexity, the target genome can be subdivided, with individual FLink analyses run across each subdivision^107^. However, each subdivision must have a suitable number of variant sites to enable effective inference of demographic parameters with 10,000 suggested as a minimum . As such, we first ran one instance of FLink for every contig in the *Pocillopora* and *Porites* genomes with at least 10,000 variants (subsampling using BCFtools v1.9^71^) using 4 cores per instance and a total of 800,000 MCMC iterations (300,000 burnin). Analysis showed that the demographic parameters converged at similar values across contigs within each of the respective genomes. To identify loci under selection in the remaining contigs (i.e. those with <10,000 variants), we therefore ran one instance of FLink per contig with parameters fixed according to the converged demographic parameters of the previous runs. Contig-wise results were consolidated and loci under divergent selection at the higher hierarchy level (i.e., between lineages; output file Posterior_A.txt) were assessed by calculating a false discovery rate (FDR) from the posterior alpha distributions following the software documentation using a custom python script. The Nextflow^109^ pipelines used to run the analyses and the Python and R scripts used to process the results are available on GitHub (https://github.com/didillysquat/TARA_host_popgen_2022).

### Supplementary information

Supplementary Material

## Data Availability

Sequence data used in this study are accessible under the Tara Pacific Umbrella BioProject PRJEB47249. More specifically, metagenomic sequencing raw data can be directly accessed under PRJEB52368. A collection of resource files including raw and filtered vcf files have been deposited on Zenodo 10.5281/zenodo.7180966^75^.

## Code availability

The code/scripts used to conduct the analyses are detailed on Zenodo 10.5281/zenodo.7180966^75^. The Nextflow pipelines used to run the analyses and the Python and R scripts used to process the results are available on GitHub (https://github.com/didillysquat/TARA_host_popgen_2022).
