# Supplementary material for: Disparate genetic divergence patterns in three corals across a pan-Pacific environmental gradient highlight species-specific adaptation
Source: NPJ Biodivers. 2023 Jul 7;2:15. doi: 10.1038/s44185-023-00020-8 (PMC11332039; doi:10.1038/s44185-023-00020-8)
Supplement: Supplementary file 1 — Supplementary Material [file 44185_2023_20_MOESM1_ESM.pdf]

SUPPLEMENTARY MATERIAL to Voolstra *et al.* 2023, npjBiodiversity

SUPPLEMENTARY FIGURES

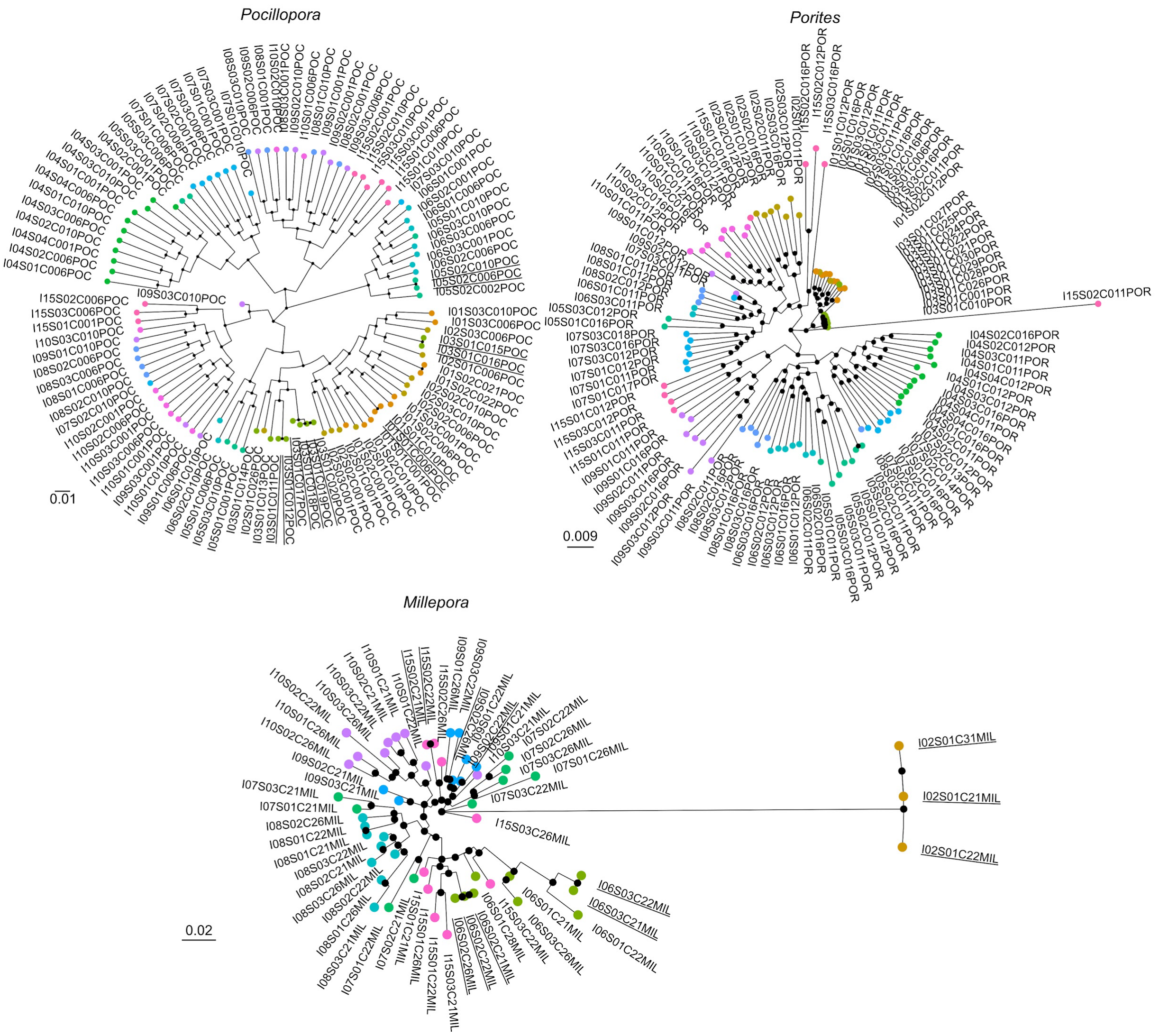

Supplementary Figure 1. Maximum likelihood phylogenetic trees for *Pocillopora*, *Porites*, and *Millepora*. Node support ≥ 80% is indicated with black circles. Leaves are colored according to the island of collection.

A

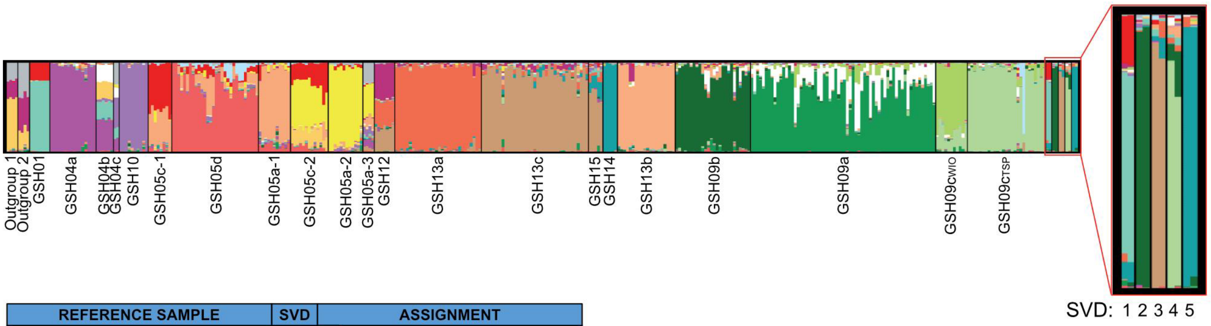

B

REFERENCE SAMPLE	SVD		ASSIGNMENT
I07S03C10	SVD1		=GSH01; = *P. effusa*
I05S02C06	SVD1		=GSH01; = *P. effusa*
I09S01C10	SVD2		=GSH09b; = *P. meandrina*
I10S03C10	SVD2		=GSH09b; = *P. meandrina*
I10S02C10	SVD3		=GSH13c; = *P. verrucosa*
I09S03C06	SVD3		=GSH13c; = *P. verrucosa*
I01S01C10	SVD4		=GSH09CTSP; = *P. grandis*
I05S03C10	SVD4		=GSH09CTSP; = *P. grandis*
I05S03C01	SVD5		=GSH14; ≈ *P. verrucosa*
I07S03C01	SVD5		=GSH14; ≈ *P. verrucosa*

Supplementary Figure 2. **Assignment of taxonomy in *Pocillopora***. A) sNMF results from the inclusion of two representative samples for each SVD lineage with the dataset of [30]. Bars show the contribution of each of 20 ancestral lineages to the samples. The ten samples from this study are on the right of the figure. Samples are grouped according to the identified lineages. B) Assignation of taxonomy to the ten reference samples. Colors correspond to the SVD lineages used in the main manuscript and the sNMF ancestral lineages used in A.

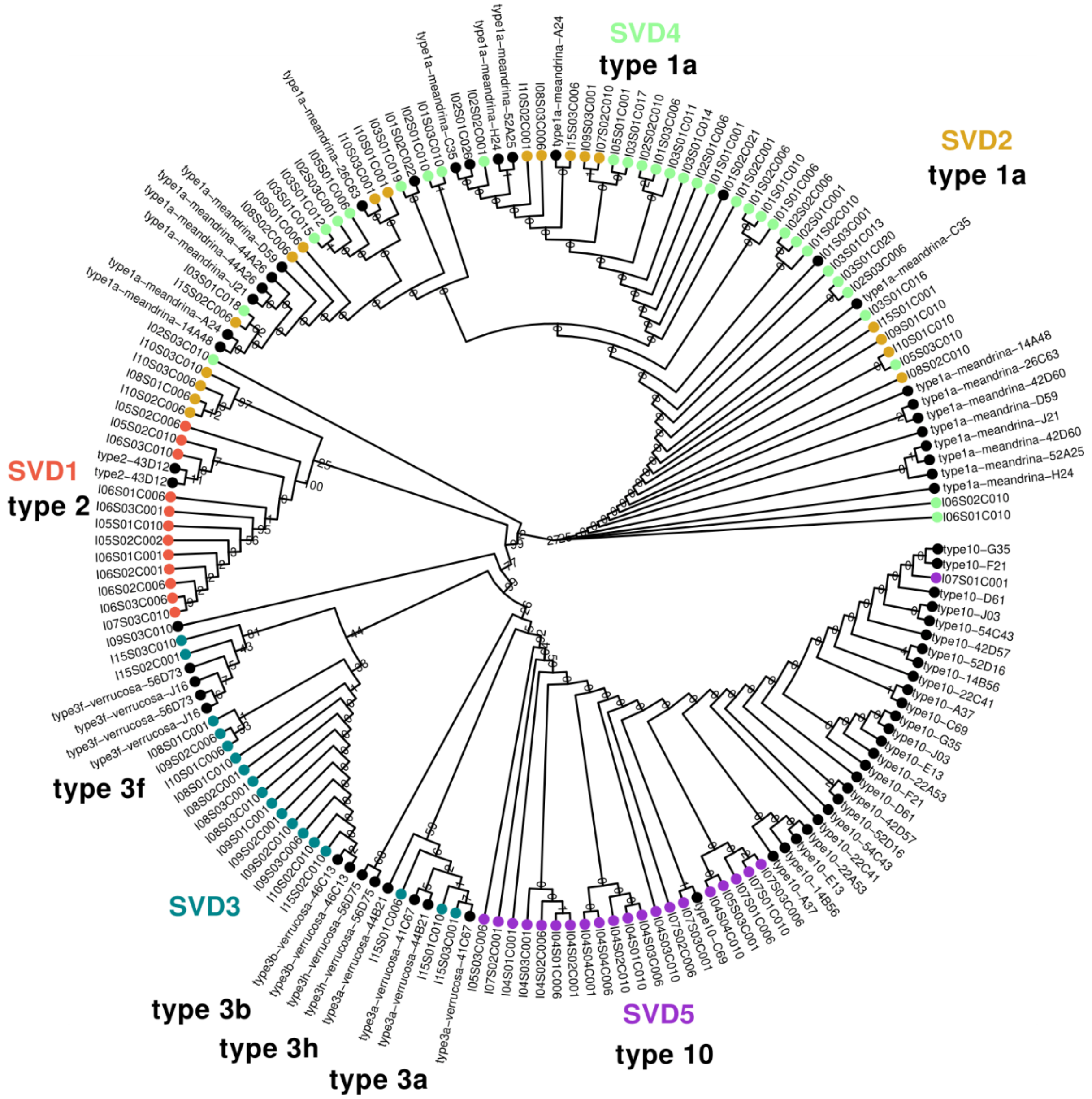

Supplementary Figure 3. mtORF-based maximum likelihood phylogeny for *Pocillopora*.

Phylogenetic analysis of the mitochondrial open reading frame (mtORF) sequences extracted from metagenomic reads in *Pocillopora*. Tip labels indicate SVD lineages identified in this study and previously reported mtORF haplotypes following Johnston *et al.* 2022.

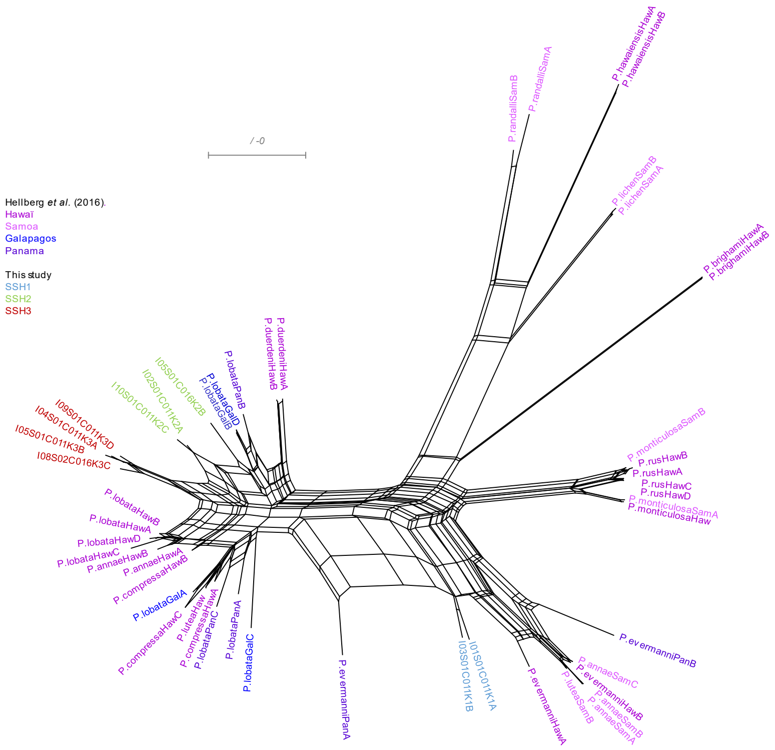

Supplementary Figure 4. Genotype network obtained from the combined POFAD analyses of three *Porites* species diagnostic genes. This network represents the genetic relationships between the reference individuals from Hellberg *et al.* (2016), colored according to their geographical origin, and the representative TARA Pacific individuals (one per subcluster within each SSH), colored according to their SSH. The three genes used were MM32, MM100 and ATPaseB, which allowed for the widest species diversity within Hellberg *et al.* (2016) dataset.

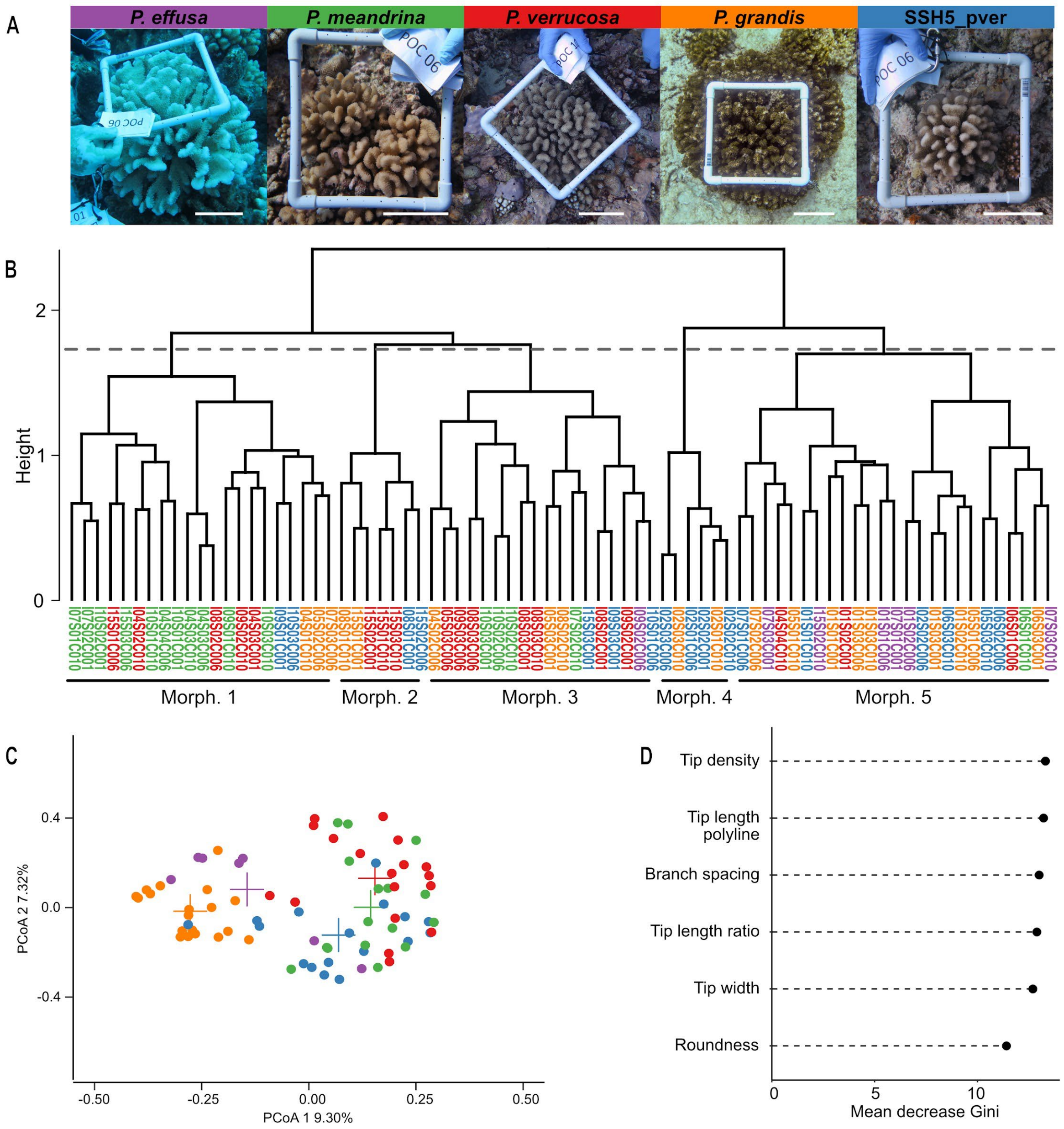

Supplementary Figure 5. Morphological analysis of *Pocillopora*. A) Representative photos for each of the five SSH. B) Dendrogram of hierarchical clustering based on unsupervised Random Forest analysis. The cutoff used to cluster the samples into five groups (morphotypes) is indicated by the horizontal gray dotted line. Morphotypes are annotated below the dendrogram. C) PCoA generated from the unsupervised Random Forest proximity matrix with samples colored according to the SVD designations given in A and with the centroids of the groups indicated as crosses. D) The mean decrease in Gini test indicating the importance of each variable in determining the homogeneity in the nodes of the decision trees in the unsupervised Random Forest model.

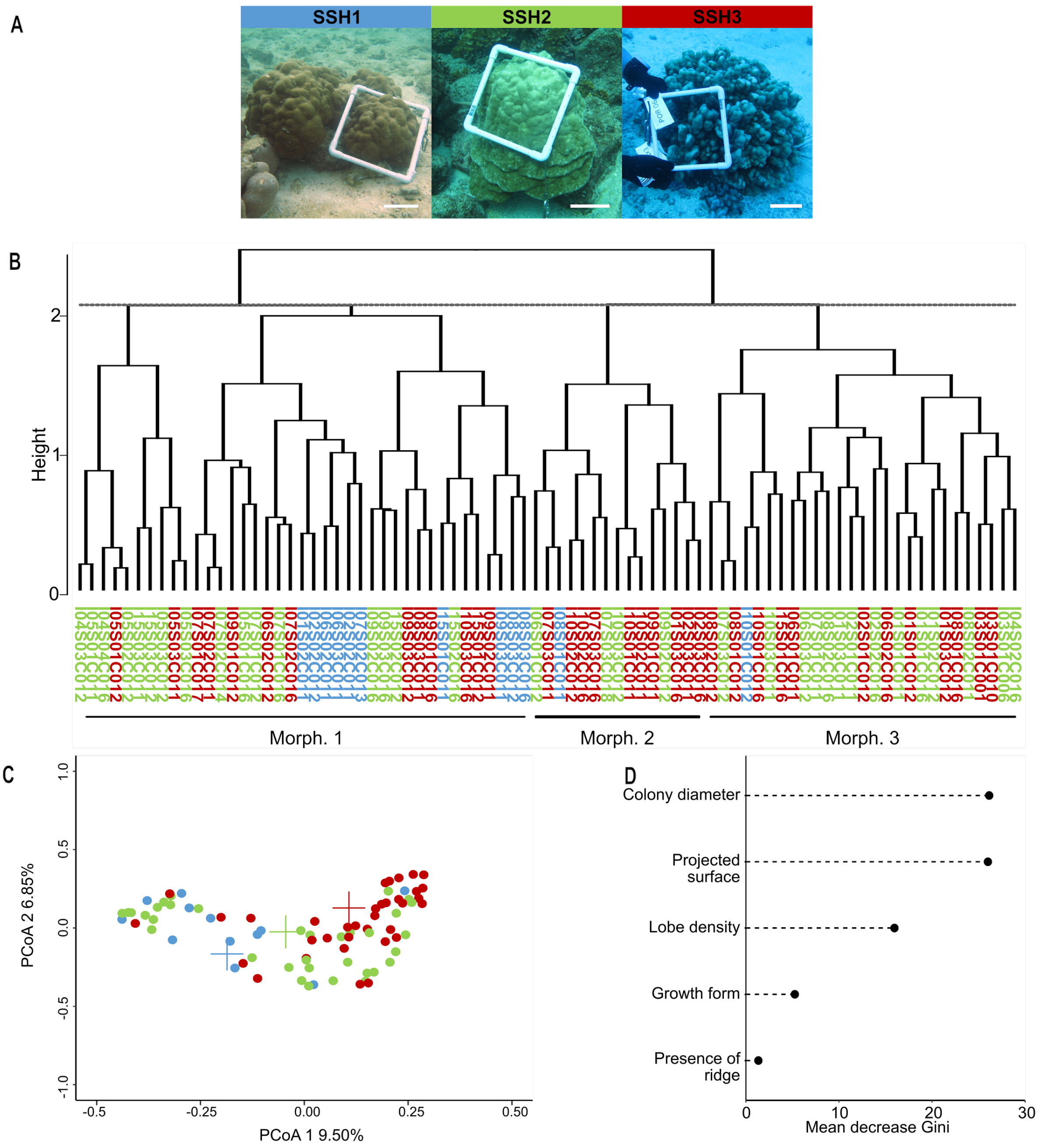

Supplementary Figure 6. Morphological analysis of *Porites*. A) Representative photos for each of the three SSH. B) Dendrogram of hierarchical clustering based on unsupervised Random Forest analysis. The cutoff used to cluster the samples into three groups (morphotypes) is indicated by the horizontal gray dotted line. Morphotypes are annotated below the dendrogram. C) PCoA generated from the unsupervised Random Forest proximity matrix with samples colored according to the sNMF designations given in A and with the centroids of the groups indicated as crosses. D) The mean decrease in Gini test indicating the importance of each variable in determining the homogeneity in the nodes of the decision trees in the unsupervised Random Forest model.

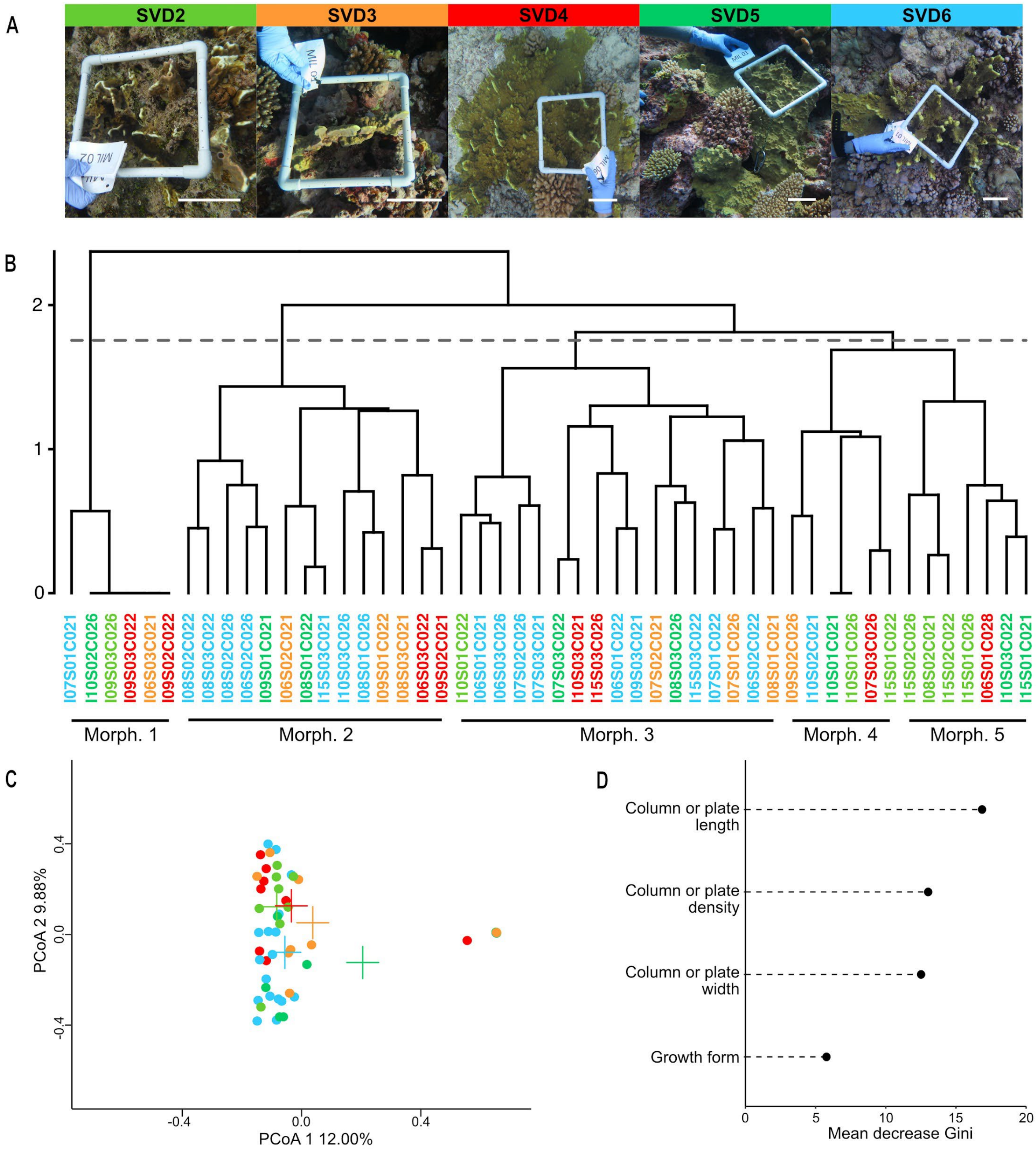

Supplementary Figure 7. Morphological analysis of *Millepora*. A) Representative photos for each of the five genetic delineations (SVD1 *M. intricata* samples excluded). B) Dendrogram of hierarchical clustering based on unsupervised Random Forest analysis. The cutoff used to cluster the samples into five groups (morphotypes) is indicated by the horizontal gray dotted line. Morphotypes are annotated below the dendrogram. C) PCoA generated from the unsupervised Random Forest proximity matrix with samples colored according to the SVD designations given in A and with the centroids of the groups indicated as crosses. D)The mean decrease in Gini test indicating the importance of each variable in determining the homogeneity in the nodes of the decision trees in the unsupervised Random Forest model.

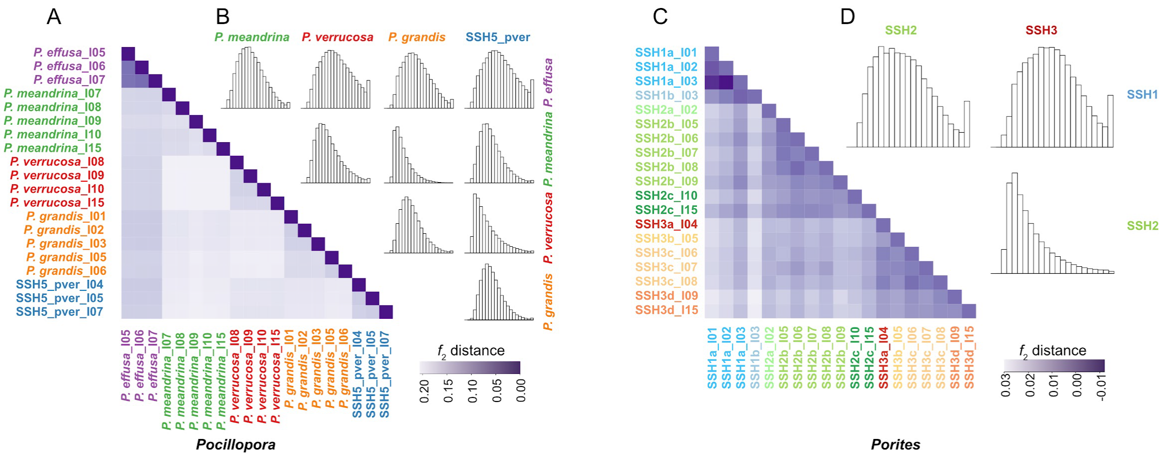

Supplementary Figure 8. Characterization of among and within SSH genetic differentiation. A & C) f_2 distances indicating correlations in genetic content between *Pocillopora* and *Porites* samples, respectively. B & D) Distributions of between SSH Weir's F_{ST} values calculated in 500 bp sliding windows for *Pocillopora* and *Porites*, respectively. For each histogram the y axis represents abundance while the x axis represents F_{ST} value with 0 on the left and 1 on the right.

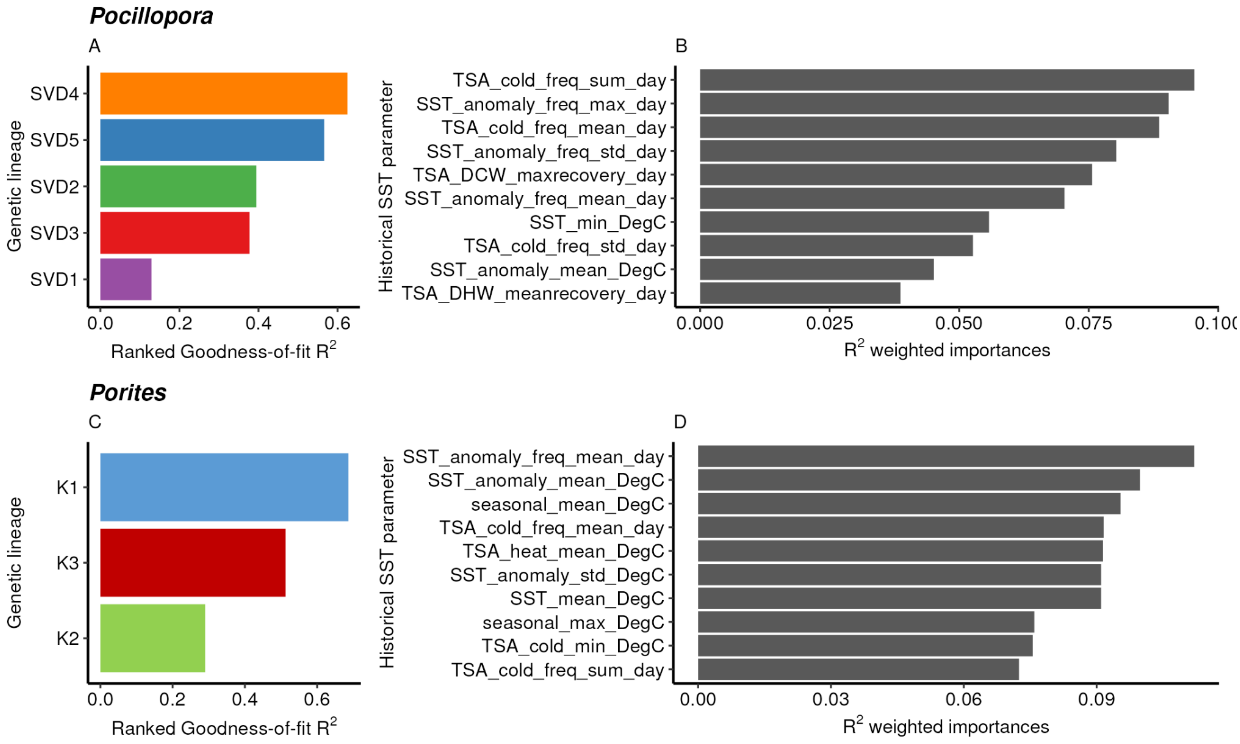

Supplementary Figure 9. Importance of the environment for the prediction of genetic lineage of *Pocillopora* and *Porites.* Performance of the gradientForest on the different genetic lineages for Pocillopora (A) and Porites (C), and the 10 most important environmental parameters for each genus (B, D).

SUPPLEMENTARY TABLES

Supplementary Table 1. Coordinates and number of samples collected at each of 32 sites from 11 islands across the Pacific.

Island number	Island name	Site number	Latitude	Longitude	*Pocillopora*	*Porites*	*Millepora*
1	Las Perlas	1	8.5795	-79.02055	3	3	0
1	Las Perlas	2	8.597875	-79.024997	5	3	0
1	Las Perlas	3	8.6501	-79.0339	3	3	0
2	Coiba	1	7.8732	-81.7942	4	3	3
2	Coiba	2	7.6513	-81.697	3	3	0
2	Coiba	3	7.2092	-81.7962	3	3	0
3	Malpelo	1	3.9873	-81.5915	10	12	0
4	Easter	1	-27.0688	-109.3233	3	3	0
4	Easter	2	-27.0674	-109.335	3	3	0
4	Easter	3	-27.1326	-109.434	3	3	0
4	Easter	4	-27.1486	-109.4442	3	3	0
5	Ducie	1	-24.6971	-124.8021	3	3	0
5	Ducie	2	-24.6971	-124.7947	3	3	0
5	Ducie	3	-24.6709	-124.7757	3	3	0
6	Gambier	1	-23.0748	-135.072	3	1	3
6	Gambier	2	-23.1651	-134.8482	3	3	3
6	Gambier	3	-23.2374	-134.9566	3	3	3
7	Moorea	1	-17.482567	-149.886467	3	4	3
7	Moorea	2	-17.518433	-149.924	3	5	3
7	Moorea	3	-17.489667	-149.75505	3	4	3
8	Aitutaki	1	-18.839967	-159.8009	3	3	3
8	Aitutaki	2	-18.913967	-159.8451	3	3	3
8	Aitutaki	3	-18.8678	-159.818667	3	3	3
9	Niue	1	-19.113433	-169.914233	3	3	3
9	Niue	2	-18.985417	-169.903267	3	3	3
9	Niue	3	-19.041817	-169.9185	3	3	3
10	Samoa	1	-14.010967	-171.843117	3	3	3
10	Samoa	2	-14.0616	-171.430367	3	3	3

10	Samoa	3	-13.918767	-171.541567	3	3	3
15	Guam	1	13.249733	144.64495	3	3	3
15	Guam	2	13.4172	144.644633	3	3	3
15	Guam	3	13.3434	144.6361	3	3	3
Totals					106	109	57

Supplementary Table 2. Testing of secondary species hypotheses (SSH) in *Pocillopora* and *Porites* using Bayes factor delimitation with genomic data (BFD*). The hypothesis resolving each SVD as a distinct species was the second most likely in each of two replicate runs (Run A and Run B) and is underlined.

Run A		Run B	
**Species split (nb. species)**	**Marginal Likelihood**	**Species split (nb. species)**	**Marginal Likelihood**
Pocillopora			
SVD1/SVD2/SVD4/SVD3-SVD5 (4)	-12963.059	SVD1/SVD2-SVD4/SVD3-SVD5 (3)	-12883.316
SVD1/SVD2/SVD3/SVD4/SVD5 (5)	-12973.734	SVD1/SVD2/SVD3/SVD4/SVD5 (5)	-12895.469
SVD1/SVD2-SVD4/SVD3-SVD5 (3)	-12979.111	SVD1/SVD2 /SVD4/SVD3-SVD5 (4)	-12902.967
SVD1/SVD2-SVD4/SVD3/SVD5 (4)	-13005.359	SVD1/SVD2-SVD4/SVD3/SVD5 (4)	-13001.632
SVD1/SVD2-SVD3-SVD4-SVD5 (2)	-13193.471	SVD1/SVD2-SVD3-SVD4-SVD5 (2)	-13075.553
Porites			
K1a/K1b/K2abc/K3abcd (4)	-9149.427	K1ab/K2a/K2b/K2c/K3abcd (5)	-9181.521
K1ab/K2abc/K3abcd (3)	-9154.839	K1ab/K2abc/K3abcd (3)	-9188.583
K1ab/K2a/K2b/K2c/K3abcd (5)	-9174.805	K1a/K1b/K2a/K2bc/K3a/K3bcd (6)	-9193.623
K1a/K1b/K2a/K2bc/K3a/K3bcd (6)	-9178.793	K1a/K1b/K2abc/K3abcd (4)	-9193.789
K1ab/K2abc/K3a/K3b/K3c/K3d (6)	-9191.031	K1ab/K2abc/K3a/K3b/K3c/K3d (6)	-9210.469

Supplementary Table 3. List of all colonies sampled and their genetic delineations. The sample names as used in the present study are given in 'sample name'. 'TARA barcode' and 'sampling-design_label' provide additional sample identifiers that integrate with the wider Tara Pacific dataset. The columns 'species' (*Pocillopora*), 'SSH,subcluster' (*Porites*), and 'sNMF,SVDquartet' (*Millepora*) designate the respective species and genetic delineations. The column 'MLL (clone)' denotes membership of the sample to a given clonal Multi Locus Lineage (repeated genet; MLL), and the column "ramets kept" marks if the colony was kept in the clonality pruned dataset (1 ramet per genet). The column BFD* (*Pocillopora* and *Porites* only) marks the replicate BFD* run to which it belongs. The "reference individual" column (*Pocillopora* and *Porites*) marks the samples used for species name attribution. The 'introgression population' column gives the population to which the colony was assigned for the introgression analysis. *NB:* In Porites, SSH1 corresponds to *P. evermanni*, and SSH2 and SSH3 are cryptic species within *P. lobata*, SSH2_plob and SSH3_plob.

sample name	TARA barcode	sampling-design_label	species	introgression population	MLL (clone)	ramets kept	BFD*	reference individual
Pocillopora								
I01S01C001POC	TARA_CO-0000002	OA000-I01-S01-C001	*P. grandis*	SVD4_I01		x		
I01S01C006POC	TARA_CO-0000019	OA000-I01-S01-C006	*P. grandis*	SVD4_I01	1_10		A	
I01S01C010POC	TARA_CO-0000032	OA000-I01-S01-C010	*P. grandis*	SVD4_I01	1_10	x	B	x
I01S02C001POC	TARA_CO-0000065	OA000-I01-S02-C001	*P. grandis*	SVD4_I01		x		
I01S02C006POC	TARA_CO-0000070	OA000-I01-S02-C006	*P. grandis*	SVD4_I01		x		
I01S02C010POC	TARA_CO-0000074	OA000-I01-S02-C010	*P. grandis*	SVD4_I01		x	A	
I01S02C021POC	TARA_CO-0000125	OA000-I01-S02-C021	*P. grandis*	SVD4_I01	1_28	x		
I01S02C022POC	TARA_CO-0000126	OA000-I01-S02-C022	*P. grandis*	SVD4_I01	1_28			
I01S03C001POC	TARA_CO-0000495	OA000-I01-S03-C001	*P. grandis*	SVD4_I01		x	B	
I01S03C006POC	TARA_CO-0000500	OA000-I01-S03-C006	*P. grandis*	SVD4_I01		x		
I01S03C010POC	TARA_CO-0000504	OA000-I01-S03-C010	*P. grandis*	SVD4_I01		x		
I02S01C001POC	TARA_CO-0000140	OA000-I02-S01-C001	*P. grandis*	SVD4_I02		x		
I02S01C006POC	TARA_CO-0000145	OA000-I02-S01-C006	*P. grandis*	SVD4_I02		x	A+B	
I02S01C010POC	TARA_CO-0000149	OA000-I02-S01-C010	*P. grandis*	SVD4_I02		x		
I02S01C026POC	TARA_CO-0000205	OA000-I02-S01-C026	*P. grandis*	SVD4_I02				
I02S02C001POC	TARA_CO-0000220	OA000-I02-S02-C001	*P. grandis*	SVD4_I02		x		
I02S02C006POC	TARA_CO-0000225	OA000-I02-S02-C006	*P. grandis*	SVD4_I02		x	B	

I02S02C010POC	TARA_CO-0000229	OA000-I02-S02-C010	*P. grandis*	SVD4_I02		x	
I02S03C001POC	TARA_CO-0000385	OA000-I02-S03-C001	*P. grandis*	SVD4_I02		x	
I02S03C006POC	TARA_CO-0000390	OA000-I02-S03-C006	*P. grandis*	SVD4_I02		x	
I02S03C010POC	TARA_CO-0000394	OA000-I02-S03-C010	*P. grandis*	SVD4_I02		x	A
I03S01C011POC	TARA_CO-0000555	OA000-I03-S01-C011	*P. grandis*	SVD4_I03	1_26		A
I03S01C012POC	TARA_CO-0000556	OA000-I03-S01-C012	*P. grandis*	SVD4_I03	1_26	x	B
I03S01C013POC	TARA_CO-0000557	OA000-I03-S01-C013	*P. grandis*	SVD4_I03		x	
I03S01C014POC	TARA_CO-0000558	OA000-I03-S01-C014	*P. grandis*	SVD4_I03		x	
I03S01C015POC	TARA_CO-0000559	OA000-I03-S01-C015	*P. grandis*	SVD4_I03	1_24	x	
I03S01C016POC	TARA_CO-0000560	OA000-I03-S01-C016	*P. grandis*	SVD4_I03	1_24		
I03S01C017POC	TARA_CO-0000561	OA000-I03-S01-C017	*P. grandis*	SVD4_I03	1_8	x	A
I03S01C018POC	TARA_CO-0000562	OA000-I03-S01-C018	*P. grandis*	SVD4_I03	1_8		
I03S01C019POC	TARA_CO-0000563	OA000-I03-S01-C019	*P. grandis*	SVD4_I03	1_8		B
I03S01C020POC	TARA_CO-0000564	OA000-I03-S01-C020	*P. grandis*	SVD4_I03	1_8		
I04S01C001POC	TARA_CO-0000580	OA000-I04-S01-C001	SSH5_pver	SVD5_I04		x	
I04S01C006POC	TARA_CO-0000585	OA000-I04-S01-C006	SSH5_pver	SVD5_I04		x	
I04S01C010POC	TARA_CO-0000589	OA000-I04-S01-C010	SSH5_pver	SVD5_I04		x	B
I04S02C001POC	TARA_CO-0000655	OA000-I04-S02-C001	SSH5_pver	SVD5_I04		x	A
I04S02C006POC	TARA_CO-0000660	OA000-I04-S02-C006	SSH5_pver	SVD5_I04		x	
I04S02C010POC	TARA_CO-0000664	OA000-I04-S02-C010	SSH5_pver	SVD5_I04		x	
I04S03C001POC	TARA_CO-0000697	OA000-I04-S03-C001	SSH5_pver	SVD5_I04		x	A
I04S03C006POC	TARA_CO-0000702	OA000-I04-S03-C006	SSH5_pver	SVD5_I04		x	
I04S03C010POC	TARA_CO-0000706	OA000-I04-S03-C010	SSH5_pver	SVD5_I04		x	
I04S04C001POC	TARA_CO-0000777	OA000-I04-S04-C001	SSH5_pver	SVD5_I04		x	B
I04S04C006POC	TARA_CO-0000782	OA000-I04-S04-C006	SSH5_pver	SVD5_I04		x	
I04S04C010POC	TARA_CO-0000786	OA000-I04-S04-C010	SSH5_pver	SVD5_I04		x	
I05S01C001POC	TARA_CO-0000817	OA000-I05-S01-C001	*P. grandis*	SVD4_I05		x	B
I05S01C006POC	TARA_CO-0000822	OA000-I05-S01-C006	*P. grandis*	SVD4_I05		x	
I05S01C010POC	TARA_CO-0000826	OA000-I05-S01-C010	*P. effusa*	SVD1_I05		x	B
I05S02C002POC	TARA_CO-0000919	OA000-I05-S02-C002	*P. effusa*	SVD1_I05		x	

I05S02C006POC	TARA_CO-0000923	OA000-I05-S02-C006	*P. effusa*	SVD1_I05	0_5	x	A	x
I05S02C010POC	TARA_CO-0000927	OA000-I05-S02-C010	*P. effusa*	SVD1_I05	0_5			
I05S03C001POC	TARA_CO-0000948	OA000-I05-S03-C001	SSH5_pver	SVD5_I05		x	B	x
I05S03C006POC	TARA_CO-0000953	OA000-I05-S03-C006	SSH5_pver	SVD5_I05		x	A	
I05S03C010POC	TARA_CO-0000957	OA000-I05-S03-C010	*P. grandis*	SVD4_I05		x	A	x
I06S01C001POC	TARA_CO-0002056	OA000-I06-S01-C001	*P. effusa*	SVD1_I06		x	B	
I06S01C006POC	TARA_CO-0002061	OA000-I06-S01-C006	*P. effusa*	SVD1_I06		x		
I06S01C010POC	TARA_CO-0002065	OA000-I06-S01-C010	*P. grandis*	SVD4_I06		x	B	
I06S02C001POC	TARA_CO-0002126	OA000-I06-S02-C001	*P. effusa*	SVD1_I06		x		
I06S02C006POC	TARA_CO-0002131	OA000-I06-S02-C006	*P. effusa*	SVD1_I06		x		
I06S02C010POC	TARA_CO-0002135	OA000-I06-S02-C010	*P. grandis*	SVD4_I06		x	A	
I06S03C001POC	TARA_CO-0002286	OA000-I06-S03-C001	*P. effusa*	SVD1_I06		x		
I06S03C006POC	TARA_CO-0002291	OA000-I06-S03-C006	*P. effusa*	SVD1_I06		x		
I06S03C010POC	TARA_CO-0002295	OA000-I06-S03-C010	*P. effusa*	SVD1_I06		x	A	
I07S01C001POC	TARA_CO-0002356	OA000-I07-S01-C001	SSH5_pver	SVD5_I07		x		
I07S01C006POC	TARA_CO-0002361	OA000-I07-S01-C006	SSH5_pver	SVD5_I07		x		
I07S01C010POC	TARA_CO-0002365	OA000-I07-S01-C010	SSH5_pver	SVD5_I07		x	B	
I07S02C001POC	TARA_CO-0002456	OA000-I07-S02-C001	SSH5_pver	SVD5_I07		x		
I07S02C006POC	TARA_CO-0002461	OA000-I07-S02-C006	SSH5_pver	SVD5_I07		x	A	
I07S02C010POC	TARA_CO-0002465	OA000-I07-S02-C010	*P. meandrina*	SVD2_I07		x	A+B	
I07S03C001POC	TARA_CO-0002566	OA000-I07-S03-C001	SSH5_pver	SVD5_I07		x		x
I07S03C006POC	TARA_CO-0002572	OA000-I07-S03-C006	SSH5_pver	SVD5_I07		x		
I07S03C010POC	TARA_CO-0002576	OA000-I07-S03-C010	*P. effusa*	SVD1_I07		x	A+B	x
I08S01C001POC	TARA_CO-0002596	OA000-I08-S01-C001	*P. verrucosa*	SVD3_I08		x	B	
I08S01C006POC	TARA_CO-0002601	OA000-I08-S01-C006	*P. meandrina*	SVD2_I08		x	B	
I08S01C010POC	TARA_CO-0002605	OA000-I08-S01-C010	*P. verrucosa*	SVD3_I08		x		
I08S02C001POC	TARA_CO-0002686	OA000-I08-S02-C001	*P. verrucosa*	SVD3_I08		x		
I08S02C006POC	TARA_CO-0002691	OA000-I08-S02-C006	*P. meandrina*	SVD2_I08		x		
I08S02C010POC	TARA_CO-0002695	OA000-I08-S02-C010	*P. meandrina*	SVD2_I08		x	A	
I08S03C001POC	TARA_OA-0002257	OA000-I08-S03-C001	*P. verrucosa*	SVD3_I08		x	A	

I08S03C006POC	TARA_OA-0002262	OA000-I08-S03-C006	*P. meandrina*	SVD2_I08	x		
I08S03C010POC	TARA_OA-0002266	OA000-I08-S03-C010	*P. verrucosa*	SVD3_I08	x		
I09S01C001POC	TARA_OA-0002323	OA000-I09-S01-C001	*P. verrucosa*	SVD3_I09	x	B	
I09S01C006POC	TARA_OA-0002328	OA000-I09-S01-C006	*P. meandrina*	SVD2_I09	x	B	
I09S01C010POC	TARA_OA-0002332	OA000-I09-S01-C010	*P. meandrina*	SVD2_I09	x		x
I09S02C001POC	TARA_FH-0000951	OA000-I09-S02-C001	*P. verrucosa*	SVD3_I09	x		
I09S02C006POC	TARA_FH-0000956	OA000-I09-S02-C006	*P. verrucosa*	SVD3_I09	x	A	
I09S02C010POC	TARA_FH-0000960	OA000-I09-S02-C010	*P. verrucosa*	SVD3_I09	x		
I09S03C001POC	TARA_OA-0002451	OA000-I09-S03-C001	*P. meandrina*	SVD2_I09	x	A	
I09S03C006POC	TARA_OA-0002456	OA000-I09-S03-C006	*P. verrucosa*	SVD3_I09	x		x
I09S03C010POC	TARA_OA-0002460	OA000-I09-S03-C010	SVD2 SVD3	SVD2 SVD3_I09	x	A+B	
I10S01C001POC	TARA_CO-0004051	OA000-I10-S01-C001	*P. meandrina*	SVD2_I10	x		
I10S01C006POC	TARA_CO-0004036	OA000-I10-S01-C006	*P. verrucosa*	SVD3_I10	x	B	
I10S01C010POC	TARA_CO-0004040	OA000-I10-S01-C010	*P. meandrina*	SVD2_I10	x	B	
I10S02C001POC	TARA_CO-0004201	OA000-I10-S02-C001	*P. meandrina*	SVD2_I10	x	A	
I10S02C006POC	TARA_CO-0004206	OA000-I10-S02-C006	*P. meandrina*	SVD2_I10	x		
I10S02C010POC	TARA_CO-0004210	OA000-I10-S02-C010	*P. verrucosa*	SVD3_I10	x	A	x
I10S03C001POC	TARA_CO-0004321	OA000-I10-S03-C001	*P. meandrina*	SVD2_I10	x		
I10S03C006POC	TARA_CO-0004326	OA000-I10-S03-C006	*P. meandrina*	SVD2_I10	x		
I10S03C010POC	TARA_CO-0004330	OA000-I10-S03-C010	*P. meandrina*	SVD2_I10	x		x
I15S01C001POC	TARA_CO-0001811	OA000-I15-S01-C001	*P. meandrina*	SVD2_I15	x	B	
I15S01C006POC	TARA_CO-0001816	OA000-I15-S01-C006	*P. verrucosa*	SVD3_I15	x	B	
I15S01C010POC	TARA_CO-0001820	OA000-I15-S01-C010	*P. verrucosa*	SVD3_I15	x		
I15S02C001POC	TARA_CO-0001659	OA000-I15-S02-C001	*P. verrucosa*	SVD3_I15	x	A	
I15S02C006POC	TARA_CO-0001664	OA000-I15-S02-C006	*P. meandrina*	SVD2_I15	x		
I15S02C010POC	TARA_CO-0001668	OA000-I15-S02-C010	*P. verrucosa*	SVD3_I15	x		
I15S03C001POC	TARA_CO-0001599	OA000-I15-S03-C001	*P. verrucosa*	SVD3_I15	x		
I15S03C006POC	TARA_CO-0001604	OA000-I15-S03-C006	*P. meandrina*	SVD2_I15	x	A	
I15S03C010POC	TARA_CO-0001608	OA000-I15-S03-C010	*P. verrucosa*	SVD3_I15	x		

sample name	TARA barcode	sampling-design_label	SSH,subcluster	introgression population	MLL (clone)	ramets kept	BFD*	reference individual
Porites								
I01S01C011POR	TARA_CO-0000035	OA000-I01-S01-C011	SSH1,K1a	K1a_I01			A	X
I01S01C012POR	TARA_CO-0000038	OA000-I01-S01-C012	SSH1,K1a	K1a_I01				
I01S01C016POR	TARA_CO-0000050	OA000-I01-S01-C016	SSH1,K1a	K1a_I01				
I01S02C011POR	TARA_CO-0000075	OA000-I01-S02-C011	SSH1,K1a	K1a_I01				
I01S02C012POR	TARA_CO-0000076	OA000-I01-S02-C012	SSH1,K1a	K1a_I01				
I01S02C016POR	TARA_CO-0000080	OA000-I01-S02-C016	SSH1,K1a	K1a_I01				
I01S03C011POR	TARA_CO-0000505	OA000-I01-S03-C011	SSH1,K1a	K1a_I01				
I01S03C012POR	TARA_CO-0000506	OA000-I01-S03-C012	SSH1,K1a	K1a_I01				
I01S03C016POR	TARA_CO-0000510	OA000-I01-S03-C016	SSH1,K1a	K1a_I01			B	
I02S01C011POR	TARA_CO-0000150	OA000-I02-S01-C011	SSH2,K2a	K2a_I02			A	X
I02S01C012POR	TARA_CO-0000151	OA000-I02-S01-C012	SSH2,K2a	K2a_I02				
I02S01C016POR	TARA_CO-0000155	OA000-I02-S01-C016	SSH1,K1a	K1a_I02			A	
I02S02C011POR	TARA_CO-0000230	OA000-I02-S02-C011	SSH2,K2a	K2a_I02				
I02S02C012POR	TARA_CO-0000231	OA000-I02-S02-C012	SSH2,K2a	K2a_I02			B	
I02S02C016POR	TARA_CO-0000235	OA000-I02-S02-C016	SSH2,K2a	K2a_I02				
I02S03C011POR	TARA_CO-0000395	OA000-I02-S03-C011	SSH1,K1a	K1a_I02			B	
I02S03C012POR	TARA_CO-0000396	OA000-I02-S03-C012	SSH2,K2a	K2a_I02			A	
I02S03C016POR	TARA_CO-0000400	OA000-I02-S03-C016	SSH2,K2a	K2a_I02			B	
I03S01C001POR	TARA_CO-0000525	OA000-I03-S01-C001	SSH1,K1b	K1b_I03			A	X
I03S01C006POR	TARA_CO-0000530	OA000-I03-S01-C006	SSH1,K1a	K1a_I03			A+B	
I03S01C010POR	TARA_CO-0000534	OA000-I03-S01-C010	SSH1,K1b	K1b_I03			B	
I03S01C021POR	TARA_CO-0000565	OA000-I03-S01-C021	SSH1,K1b	K1b_I03				
I03S01C022POR	TARA_CO-0000566	OA000-I03-S01-C022	SSH1,K1b	K1b_I03				
I03S01C024POR	TARA_CO-0000568	OA000-I03-S01-C024	SSH1,K1b	K1b_I03				
I03S01C025POR	TARA_CO-0000569	OA000-I03-S01-C025	SSH1,K1b	K1b_I03				
I03S01C026POR	TARA_CO-0000570	OA000-I03-S01-C026	SSH1,K1b	K1b_I03				
I03S01C027POR	TARA_CO-0000571	OA000-I03-S01-C027	SSH1,K1b	K1b_I03				

I03S01C028POR	TARA_CO-0000572	OA000-I03-S01-C028	SSH1,K1b	K1b_I03		
I03S01C029POR	TARA_CO-0000573	OA000-I03-S01-C029	SSH1,K1b	K1b_I03	A	
I03S01C030POR	TARA_CO-0000574	OA000-I03-S01-C030	SSH1,K1b	K1b_I03	B	
I04S01C011POR	TARA_CO-0000590	OA000-I04-S01-C011	SSH3,K3a	K3a_I04	A	X
I04S01C012POR	TARA_CO-0000591	OA000-I04-S01-C012	SSH3,K3a	K3a_I04	B	
I04S01C016POR	TARA_CO-0000595	OA000-I04-S01-C016	SSH3,K3a	K3a_I04		
I04S02C011POR	TARA_CO-0000675	OA000-I04-S02-C011	SSH3,K3a	K3a_I04		
I04S02C012POR	TARA_CO-0000676	OA000-I04-S02-C012	SSH3,K3a	K3a_I04		
I04S02C016POR	TARA_CO-0000680	OA000-I04-S02-C016	SSH3,K3a	K3a_I04		
I04S03C011POR	TARA_CO-0000727	OA000-I04-S03-C011	SSH3,K3a	K3a_I04		
I04S03C012POR	TARA_CO-0000728	OA000-I04-S03-C012	SSH3,K3a	K3a_I04		
I04S03C016POR	TARA_CO-0000732	OA000-I04-S03-C016	SSH3,K3a	K3a_I04		
I04S04C011POR	TARA_CO-0000787	OA000-I04-S04-C011	SSH3,K3a	K3a_I04	A	
I04S04C012POR	TARA_CO-0000788	OA000-I04-S04-C012	SSH3,K3a	K3a_I04		
I04S04C016POR	TARA_CO-0000792	OA000-I04-S04-C016	SSH3,K3a	K3a_I04	B	
I05S01C011POR	TARA_CO-0000797	OA000-I05-S01-C011	SSH3,K3b	K3b_I05	A	X
I05S01C012POR	TARA_CO-0000798	OA000-I05-S01-C012	SSH3,K3b	K3b_I05	B	
I05S01C016POR	TARA_CO-0000802	OA000-I05-S01-C016	SSH2,K2b	K2b_I05	A	X
I05S02C011POR	TARA_CO-0000908	OA000-I05-S02-C011	SSH3,K3b	K3b_I05		
I05S02C012POR	TARA_CO-0000909	OA000-I05-S02-C012	SSH3,K3b	K3b_I05		
I05S02C016POR	TARA_CO-0000913	OA000-I05-S02-C016	SSH3,K3b	K3b_I05		
I05S03C011POR	TARA_CO-0000958	OA000-I05-S03-C011	SSH3,K3b	K3b_I05	A	
I05S03C012POR	TARA_CO-0000959	OA000-I05-S03-C012	SSH2,K2b	K2b_I05	B	
I05S03C016POR	TARA_CO-0000963	OA000-I05-S03-C016	SSH3,K3b	K3b_I05	B	
I06S01C011POR	TARA_CO-0002046	OA000-I06-S01-C011	SSH2,K2b	K2b_I06	A	
I06S01C012POR	TARA_CO-0002047	OA000-I06-S01-C012	SSH3,K3c	K3c_I06	A	
I06S01C016POR	TARA_CO-0002051	OA000-I06-S01-C016	SSH3,K3c	K3c_I06		
I06S02C011POR	TARA_CO-0002136	OA000-I06-S02-C011	SSH3,K3c	K3c_I06		
I06S02C012POR	TARA_CO-0002137	OA000-I06-S02-C012	SSH3,K3c	K3c_I06		
I06S02C016POR	TARA_CO-0002141	OA000-I06-S02-C016	SSH3,K3c	K3c_I06		

I06S03C011POR	TARA_CO-0002276	OA000-I06-S03-C011	SSH2,K2b	K2b_I06	B	
I06S03C012POR	TARA_CO-0002277	OA000-I06-S03-C012	SSH3,K3c	K3c_I06	B	
I06S03C016POR	TARA_CO-0002281	OA000-I06-S03-C016	SSH3,K3c	K3c_I06		
I07S01C011POR	TARA_CO-0002366	OA000-I07-S01-C011	SSH2,K2b	K2b_I07	B	
I07S01C012POR	TARA_CO-0002367	OA000-I07-S01-C012	SSH2,K2b	K2b_I07		
I07S01C016POR	TARA_CO-0002371	OA000-I07-S01-C016	SSH3,K3c	K3c_I07	B	
I07S01C017POR	TARA_CO-0002372	OA000-I07-S01-C017	SSH2,K2b	K2b_I07		
I07S02C011POR	TARA_CO-0002446	OA000-I07-S02-C011	SSH3,K3c	K3c_I07		
I07S02C012POR	TARA_CO-0002447	OA000-I07-S02-C012	SSH3,K3c	K3c_I07		
I07S02C013POR	TARA_CO-0002448	OA000-I07-S02-C013	SSH3,K3c	K3c_I07		
I07S02C014POR	TARA_CO-0002449	OA000-I07-S02-C014	SSH3,K3c	K3c_I07	A	
I07S02C016POR	TARA_CO-0002451	OA000-I07-S02-C016	SSH3,K3c	K3c_I07		
I07S03C011POR	TARA_CO-0002577	OA000-I07-S03-C011	SSH2,K2b	K2b_I07		
I07S03C012POR	TARA_CO-0002578	OA000-I07-S03-C012	SSH2,K2b	K2b_I07		
I07S03C016POR	TARA_CO-0002582	OA000-I07-S03-C016	SSH2,K2b	K2b_I07		
I07S03C018POR	TARA_CO-0002584	OA000-I07-S03-C018	SSH2,K2b	K2b_I07	A	
I08S01C011POR	TARA_CO-0002606	OA000-I08-S01-C011	SSH2,K2b	K2b_I08	A	
I08S01C012POR	TARA_CO-0002607	OA000-I08-S01-C012	SSH2,K2b	K2b_I08		
I08S01C016POR	TARA_CO-0002611	OA000-I08-S01-C016	SSH3,K3c	K3c_I08	A	
I08S02C011POR	TARA_CO-0002696	OA000-I08-S02-C011	SSH3,K3c	K3c_I08		
I08S02C012POR	TARA_CO-0002697	OA000-I08-S02-C012	SSH2,K2b	K2b_I08	B	
I08S02C016POR	TARA_CO-0002701	OA000-I08-S02-C016	SSH3,K3c	K3c_I08		X
I08S03C011POR	TARA_OA-0002267	OA000-I08-S03-C011	SSH3,K3c	K3c_I08		
I08S03C012POR	TARA_OA-0002268	OA000-I08-S03-C012	SSH3,K3c	K3c_I08		
I08S03C016POR	TARA_OA-0002272	OA000-I08-S03-C016	SSH3,K3c	K3c_I08	B	
I09S01C011POR	TARA_OA-0002333	OA000-I09-S01-C011	SSH3,K3d	K3d_I09	A	X
I09S01C012POR	TARA_OA-0002334	OA000-I09-S01-C012	SSH2,K2b	K2b_I09	A	
I09S01C016POR	TARA_OA-0002338	OA000-I09-S01-C016	SSH3,K3d	K3d_I09		
I09S02C011POR	TARA_FH-0000961	OA000-I09-S02-C011	SSH3,K3d	K3d_I09		
I09S02C012POR	TARA_FH-0000962	OA000-I09-S02-C012	SSH2,K2b	K2b_I09	B	

I09S02C016POR	TARA_FH-0000966	OA000-I09-S02-C016	SSH3,K3d	K3d_I09		
I09S03C011POR	TARA_OA-0002461	OA000-I09-S03-C011	SSH3,K3d	K3d_I09		
I09S03C012POR	TARA_OA-0002462	OA000-I09-S03-C012	SSH3,K3d	K3d_I09		
I09S03C016POR	TARA_OA-0002466	OA000-I09-S03-C016	SSH3,K3d	K3d_I09	B	
I10S01C011POR	TARA_CO-0004041	OA000-I10-S01-C011	SSH2,K2c	K2c_I10	A	X
I10S01C012POR	TARA_CO-0004042	OA000-I10-S01-C012	SSH2,K2c	K2c_I10		
I10S01C016POR	TARA_CO-0004046	OA000-I10-S01-C016	SSH2,K2c	K2c_I10		
I10S02C011POR	TARA_CO-0004211	OA000-I10-S02-C011	SSH2,K2c	K2c_I10		
I10S02C012POR	TARA_CO-0004212	OA000-I10-S02-C012	SSH2,K2c	K2c_I10		
I10S02C016POR	TARA_CO-0004216	OA000-I10-S02-C016	SSH2,K2c	K2c_I10		
I10S03C011POR	TARA_CO-0004331	OA000-I10-S03-C011	SSH2,K2c	K2c_I10		
I10S03C012POR	TARA_CO-0004332	OA000-I10-S03-C012	SSH2,K2c	K2c_I10		
I10S03C016POR	TARA_CO-0004336	OA000-I10-S03-C016	SSH2,K2c	K2c_I10	B	
I15S01C011POR	TARA_CO-0001821	OA000-I15-S01-C011	SSH3,K3d	K3d_I15	A	
I15S01C012POR	TARA_CO-0001822	OA000-I15-S01-C012	SSH3,K3d	K3d_I15		
I15S01C016POR	TARA_CO-0001826	OA000-I15-S01-C016	SSH2,K2c	K2c_I15	A	
I15S02C011POR	TARA_CO-0001669	OA000-I15-S02-C011	K2/K3,K2/K3	K2/K3_I15	A+B	
I15S02C012POR	TARA_CO-0001670	OA000-I15-S02-C012	SSH2,K2c	K2c_I15		
I15S02C016POR	TARA_CO-0001674	OA000-I15-S02-C016	SSH2,K2c	K2c_I15	B	
I15S03C011POR	TARA_CO-0001609	OA000-I15-S03-C011	SSH3,K3d	K3d_I15		
I15S03C012POR	TARA_CO-0001610	OA000-I15-S03-C012	SSH3,K3d	K3d_I15	B	
I15S03C016POR	TARA_CO-0001614	OA000-I15-S03-C016	SSH2,K2c	K2c_I15	A+B	

sample name	TARA barcode	sampling-design_label	sNMF,SVDquartet	introgression population	MLL (clone)	ramets kept
Millepora						
I02S01C021MIL	TARA_CO-0000200	OA000-I02-S01-C021	K4,SVD1	I02	1_54	x
I02S01C022MIL	TARA_CO-0000201	OA000-I02-S01-C022	K4,SVD1	I02	1_54	x
I02S01C031MIL	TARA_CO-0000302	OA000-I02-S01-C031	K4,SVD1	I02	1_54	x
I06S01C021MIL	TARA_CO-0002036	OA000-I06-S01-C021	K2,SVD6	I06		x
I06S01C022MIL	TARA_CO-0002037	OA000-I06-S01-C022	K2,SVD6	I06		x

I06S01C028MIL	TARA_CO-0002043	OA000-I06-S01-C028	K2,SVD6	I06		x
I06S02C021MIL	TARA_CO-0002176	OA000-I06-S02-C021	K2,SVD6	I06	1_46	
I06S02C022MIL	TARA_CO-0002177	OA000-I06-S02-C022	K2,SVD6	I06	1_46	x
I06S02C026MIL	TARA_CO-0002181	OA000-I06-S02-C026	K2,SVD6	I06	1_46	
I06S03C021MIL	TARA_CO-0002266	OA000-I06-S03-C021	K2,SVD6	I06	1_21	x
I06S03C022MIL	TARA_CO-0002267	OA000-I06-S03-C022	K2,SVD6	I06	1_21	
I06S03C026MIL	TARA_CO-0002271	OA000-I06-S03-C026	K2,SVD6	I06		x
I07S01C021MIL	TARA_CO-0002376	OA000-I07-S01-C021	K3,SVD4	I07		x
I07S01C022MIL	TARA_CO-0002377	OA000-I07-S01-C022	K3,SVD4	I07		x
I07S01C026MIL	TARA_CO-0002381	OA000-I07-S01-C026	K3,SVD4	I07		x
I07S02C021MIL	TARA_CO-0002426	OA000-I07-S02-C021	K3,SVD4	I07		x
I07S02C022MIL	TARA_CO-0002427	OA000-I07-S02-C022	K3,SVD4	I07		x
I07S02C026MIL	TARA_CO-0002431	OA000-I07-S02-C026	K3,SVD4	I07		x
I07S03C021MIL	TARA_CO-0002587	OA000-I07-S03-C021	K3,SVD4	I07		x
I07S03C022MIL	TARA_CO-0002588	OA000-I07-S03-C022	K3,SVD4	I07		x
I07S03C026MIL	TARA_CO-0002592	OA000-I07-S03-C026	K3,SVD4	I07		x
I08S01C021MIL	TARA_CO-0002617	OA000-I08-S01-C021	K2,SVD6	I08		x
I08S01C022MIL	TARA_CO-0002618	OA000-I08-S01-C022	K2,SVD6	I08		x
I08S01C026MIL	TARA_CO-0002622	OA000-I08-S01-C026	K2,SVD6	I08		x
I08S02C021MIL	TARA_CO-0002706	OA000-I08-S02-C021	K2,SVD6	I08		x
I08S02C022MIL	TARA_CO-0002707	OA000-I08-S02-C022	K2,SVD6	I08		x
I08S02C026MIL	TARA_CO-0002711	OA000-I08-S02-C026	K2,SVD6	I08		x
I08S03C021MIL	TARA_OA-0002277	OA000-I08-S03-C021	K2,SVD6	I08		x
I08S03C022MIL	TARA_OA-0002278	OA000-I08-S03-C022	K2,SVD6	I08		x
I08S03C026MIL	TARA_OA-0002282	OA000-I08-S03-C026	K2,SVD6	I08		x
I09S01C021MIL	TARA_OA-0002343	OA000-I09-S01-C021	K1/K2,SVD5	I09		x
I09S01C022MIL	TARA_OA-0002344	OA000-I09-S01-C022	K1/K2,SVD5	I09	1_33	x
I09S01C026MIL	TARA_OA-0002348	OA000-I09-S01-C026	K1/K2,SVD5	I09	1_33	
I09S02C021MIL	TARA_FH-0000971	OA000-I09-S02-C021	K1/K2,SVD5	I09		x
I09S02C022MIL	TARA_FH-0000972	OA000-I09-S02-C022	K1/K2,SVD5	I09		x
I09S02C026MIL	TARA_FH-0000976	OA000-I09-S02-C026	K1/K2,SVD5	I09		x
I09S03C021MIL	TARA_OA-0002471	OA000-I09-S03-C021	K1/K2,SVD5	I09		x
I09S03C022MIL	TARA_OA-0002472	OA000-I09-S03-C022	K1/K2,SVD5	I09		x
I10S01C021MIL	TARA_CO-0004031	OA000-I10-S01-C021	K1/K3/K2,SVD3	I10		x
I10S01C022MIL	TARA_CO-0004032	OA000-I10-S01-C022	K1/K3/K2,SVD3	I10		x

I10S01C026MIL	TARA_CO-0004056	OA000-I10-S01-C026	K1/K3/K2,SVD3	I10		x
I10S02C021MIL	TARA_CO-0004221	OA000-I10-S02-C021	K1/K3/K2,SVD3	I10		x
I10S02C022MIL	TARA_CO-0004222	OA000-I10-S02-C022	K1/K3/K2,SVD3	I10		x
I10S02C026MIL	TARA_CO-0004226	OA000-I10-S02-C026	K1/K3/K2,SVD3	I10		x
I10S03C021MIL	TARA_CO-0004341	OA000-I10-S03-C021	K1/K3/K2,SVD3	I10		x
I10S03C022MIL	TARA_CO-0004342	OA000-I10-S03-C022	K1/K3/K2,SVD3	I10		x
I10S03C026MIL	TARA_CO-0004346	OA000-I10-S03-C026	K1/K3/K2,SVD3	I10		x
I15S01C021MIL	TARA_CO-0001831	OA000-I15-S01-C021	K1,SVD2	I15		x
I15S01C022MIL	TARA_CO-0001832	OA000-I15-S01-C022	K1,SVD2	I15		x
I15S01C026MIL	TARA_CO-0001836	OA000-I15-S01-C026	K1,SVD2	I15		x
I15S02C021MIL	TARA_CO-0001679	OA000-I15-S02-C021	K1,SVD2	I15	1_40	
I15S02C022MIL	TARA_CO-0001682	OA000-I15-S02-C022	K1,SVD2	I15	1_40	x
I15S02C026MIL	TARA_CO-0001685	OA000-I15-S02-C026	K1,SVD2	I15		x
I15S03C021MIL	TARA_CO-0001619	OA000-I15-S03-C021	K1,SVD2	I15		x
I15S03C022MIL	TARA_CO-0001620	OA000-I15-S03-C022	K1,SVD2	I15		x
I15S03C026MIL	TARA_CO-0001624	OA000-I15-S03-C026	K1,SVD2	I15		x

Supplementary Table 4. Species attribution to Pocillopora genetic lineages and correspondence with previously published studies. Each SSH was attributed a species name by mapping available raw metagenomic reads onto the *Pocillopora* reference target-capture sequences from [30] to then call the species-diagnostic SNPs generated therein, and further confirmed using mtORF sequences extracted from our metagenomic data and compared to previously published records (Gélin et al., 2017; Pinzón et al., 2013; Schmidt-Roach et al., 2014; Johnston et al 2022).

This study	Oury et al. 2022 Clade/GSH	Oury et al. 2022 mtORF	Pinzón et al. 2013	Marti-Puig et al. 2014	Schmidt-Roach et al. 2014	Johnston et al 2022 mtORF	Species name
SVD1	GSH01	01	2	IIIa	n/a	2	*P.* cf *effusa*
SVD2	GSH09b	23,27	1a, 8a	IIb	e/m	1a	*P. meandrina*
SVD3	GSH13c	35,42,47, 53,54	3b, 3d, 3f, 3h	IIa	γ	3a, 3b, 3f, 3h	*P. verrucosa*
SVD4	GSH09ctsp	27	1a, 8a	IIb	e/m	1a	*P. grandis*
SVD5	GSH14	50	n/a	n/a	n/a	10	*P. verrucosa*

N.B.: N.B.: The SVD3 and SVD5 lineages constitute cryptic species within P. verrucosa from here-detailed, Oury et al. (2023), and Johnston et al 2022 analyses. P. grandis was formerly designated as P. eydouxi.

Supplementary Table 5. **Mantel tests of genetic distances against geographic and past temperature distances**. Tests are given for each of the genera and for each of the lineages within the genera. Tests for correlation between geographic and past temperature distances for each of the genera are also given. Significant ($p<0.05$) results are underlined.

	Distance	Mantel's r	p value
**Genera**			
Pocillopora	geographic	-0.019	0.517
	temperature	-0.044	0.568
Porites	geographic	0.397	0.005
	temperature	0.361	0.020
Millepora	geographic	0.722	0.053
	temperature	0.930	0.019
**Species**			
Pocillopora			
P. effusa	geographic	-0.376	0.667
	temperature	-0.577	0.833
P. meandrina	geographic	0.514	0.167
	temperature	-0.229	0.683
P. verrucosa	geographic	0.772	0.083
	temperature	-0.661	1.000
P. grandis	geographic	0.722	0.083
	temperature	-0.103	0.650
SSH5_pver	geographic	0.677	0.333
	temperature	0.816	0.333
Porites			
SSH1/*P.evermanni*	geographic	0.982	0.167
	temperature	-0.924	1.000
SSH2_plob	geographic	0.585	0.036
	temperature	0.641	0.010
SSH3_plob	geographic	0.591	0.008
	temperature	0.372	0.052
